# The E2F4/p130 Repressor Complex Cooperates with Oncogenic ΔNp73α To Inhibit Gene Expression in Human Papillomavirus 38 E6/E7-Transformed Keratinocytes and in Cancer Cells

**DOI:** 10.1128/msphere.00056-23

**Published:** 2023-03-08

**Authors:** Valerio Taverniti, Hanna Krynska, Assunta Venuti, Marie-Laure Straub, Cécilia Sirand, Eugenie Lohmann, Maria Carmen Romero-Medina, Stefano Moro, Alexis Robitaille, Luc Negroni, Denise Martinez-Zapien, Murielle Masson, Massimo Tommasino, Katia Zanier

**Affiliations:** a International Agency for Research on Cancer (IARC), World Health Organization, Lyon, France; b Biotechnology and Cell Signaling (CNRS/Université de Strasbourg, UMR 7242), Ecole Superieure de Biotechnologie de Strasbourg, Boulevard Sébastien Brant, Illkirch, France; c Proteomics platform, Institut de Génétique et de Biologie Moléculaire et Cellulaire (IGBMC)/INSERM U964/CNRS UMR 7104/Université de Strasbourg, Illkirch, France; d IRCCS Istituto Tumori Giovanni Paolo II of Bari, Bari, Italy; Northwestern University

**Keywords:** p53, ΔNp73 isoforms, E2F4, p130, protein-protein interactions, gene expression, transformation

## Abstract

Tumor suppressor p53 and its related proteins, p63 and p73, can be synthesized as multiple isoforms lacking part of the N- or C-terminal regions. Specifically, high expression of the ΔNp73α isoform is notoriously associated with various human malignancies characterized by poor prognosis. This isoform is also accumulated by oncogenic viruses, such as Epstein–Barr virus (EBV), as well as genus beta human papillomaviruses (HPV) that appear to be involved in carcinogenesis. To gain additional insight into ΔNp73α mechanisms, we have performed proteomics analyses using human keratinocytes transformed by the E6 and E7 proteins of the beta-HPV type 38 virus as an experimental model (38HK). We find that ΔNp73α associates with the E2F4/p130 repressor complex through a direct interaction with E2F4. This interaction is favored by the N-terminal truncation of p73 characteristic of ΔNp73 isoforms. Moreover, it is independent of the C-terminal splicing status, suggesting that it could represent a general feature of ΔNp73 isoforms (α, β, γ, δ, ε, ζ, θ, η, and η1). We show that the ΔNp73α-E2F4/p130 complex inhibits the expression of specific genes, including genes encoding for negative regulators of proliferation, both in 38HK and in HPV-negative cancer-derived cell lines. Such genes are not inhibited by E2F4/p130 in primary keratinocytes lacking ΔNp73α, indicating that the interaction with ΔNp73α rewires the E2F4 transcriptional program. In conclusion, we have identified and characterized a novel transcriptional regulatory complex with potential implications in oncogenesis.

**IMPORTANCE** The TP53 gene is mutated in about 50% of human cancers. In contrast, the TP63 and TP73 genes are rarely mutated but rather expressed as ΔNp63 and ΔNp73 isoforms in a wide range of malignancies, where they act as p53 antagonists. Accumulation of ΔNp63 and ΔNp73, which is associated with chemoresistance, can result from infection by oncogenic viruses such as EBV or HPV. Our study focuses on the highly carcinogenic ΔNp73α isoform and uses a viral model of cellular transformation. We unveil a physical interaction between ΔNp73α and the E2F4/p130 complex involved in cell cycle control, which rewires the E2F4/p130 transcriptional program. Our work shows that ΔNp73 isoforms can establish interactions with proteins that do not bind to the TAp73α tumor suppressor. This situation is analogous to the gain-of-function interactions of p53 mutants supporting cellular proliferation.

## INTRODUCTION

The p53 family of proteins plays a key role in cancer prevention. It consists of 3 homologous transcription factors (TFs), namely, p53, p63, and p73, which bind to common DNA promoter sites (p53RE). The TP53, TP63, and TP73 genes can be expressed as multiple isoforms lacking part of the N- or the C-terminal regions. In the case of TP73, the full-length TAp73 protein is transcribed from the P1 promoter upstream of exon 1, whereas ΔNp73 isoforms are generated from the P2 promoter within intron 3. These isoforms lack the N-terminal transactivation domain (TAD) and, consequently, act as dominant negative inhibitors of full-length TAp73 and of its TAp53 and TAp63 homologues (1–3). Interestingly, the ΔN P2 promoter contains a p53 response element (RE) that enables TAp53 and TAp73 to induce expression of ΔN isoforms (4), thus creating a negative feedback loop that fine-tunes p53 and p73 functions. Additional isoforms of TAp73 and ΔNp73 proteins (i.e., α, β, γ, δ, ε, ζ, θ, η, and η1 isoforms) are generated by alternative splicing within the 3′ region of the gene (exons 10 to 14), giving rise to differences in the C-terminal regions of the proteins (5, 6).

Expression of the TA and ΔN proteins is highly cell- and tissue-specific. Whereas the ΔN isoforms support proliferation, the TA proteins promote cell cycle arrest, senescence, and apoptosis, suggesting that the ratio between TA and ΔN proteins determines cell fate and oncogenesis (7). In healthy cells, where ΔNp73 levels are low, c-Abl phosphorylation coupled to Pin1 isomerase binding results in the stabilization of TAp73 (8–10), whereas ΔNp73 isoforms are rapidly degraded by other ubiquitin ligases that can discriminate between TA and ΔN isoforms (11) or via the calpain (12) and 20S proteasome (13) pathways.

Mutation of the TP53 gene is the most frequent genetic alteration, present in about 50% of human cancers. In contrast, the TP63 and TP73 genes are rarely mutated but are rather expressed as ΔNp63 and ΔNp73 isoforms in a wide range of human malignancies, which display unfavorable prognosis due to increased drug resistance (7, 14, 15). In particular, the ΔNp73α isoform, which comprises an intact (unspliced) C-terminal region, is upregulated in several cancers harboring wild-type TP53 and TP73 genes (including breast, prostate, liver, lung, and thyroid cancer), where it inhibits drug-induced apoptosis (15). Although the association of ΔNp73α with elevated carcinogenicity and chemoresistance can be explained by alterations of p53-regulated gene expression, it is not yet clear whether ΔNp73α could use additional mechanisms in promoting cellular transformation. Previous independent studies have shown that ΔNp73α is accumulated upon infection with the well-established oncogenic viruses, Epstein-Barr virus (EBV) (16) and other herpesviruses, such as the human cytomegalovirus (17). Accumulation of the ΔNp73α isoform is also induced in *in vitro* and *in vivo* experimental models by cutaneous beta (β) human papillomavirus (HPV) types (18–20), which appear to be involved in the development of skin squamous cell carcinoma (21).

In this study, we searched for protein factors that modulate the functions of ΔNp73α in transformed cells. We identified and characterized a novel interaction of ΔNp73α with the E2F4/p130 transcriptional repressor complex that modulates the cellular gene expression in *in vitro* HPV38 E6/E7-transformed human keratinocytes (38HK), as well as in cancer-derived cells.

## RESULTS

### ΔNp73α associates with the E2F4/p130 transcriptional repressor complex in 38HK.

**FIG 1:**
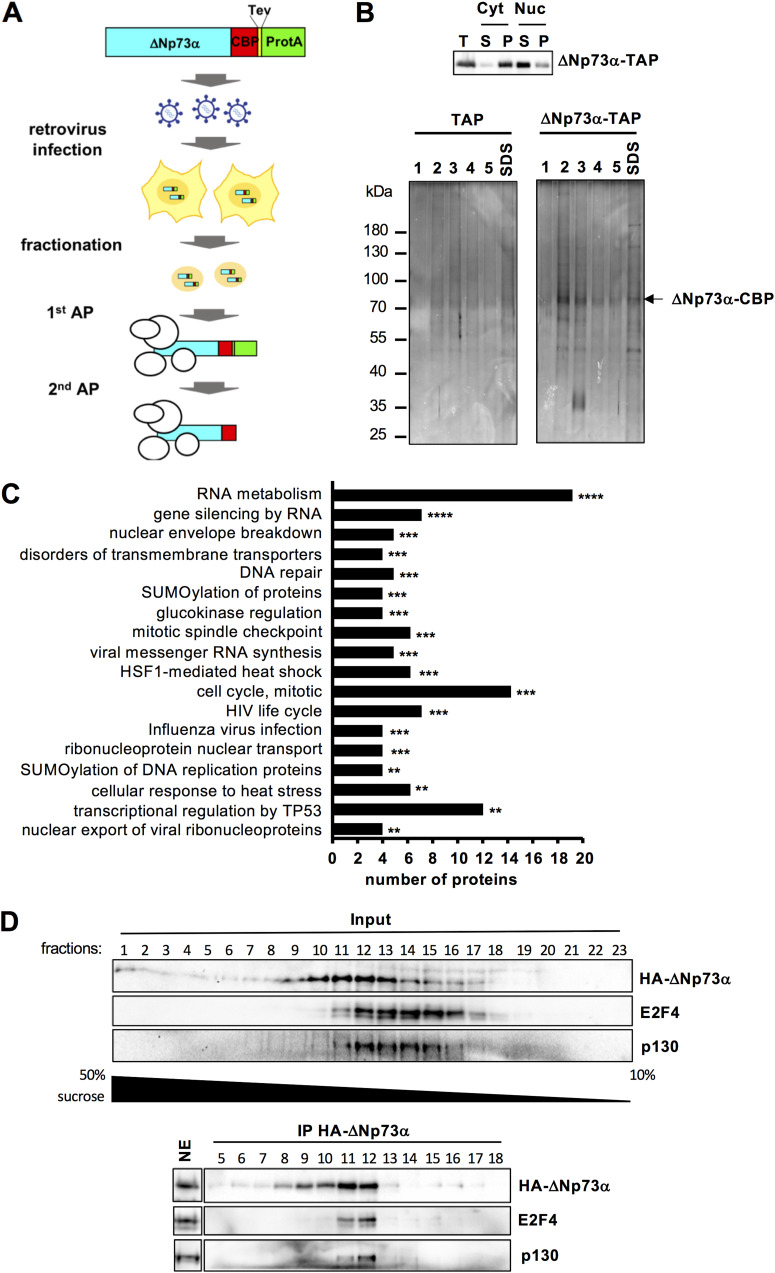
Proteomics analyses in 38HK identify the E2F4/p130 complex as a partner of ΔNp73α. (A) Illustration of the proteomics approach used to identify nuclear binding partners of ΔNp73α. (B) Expression and purification of ΔNp73α-TAP complexes. (B, upper panel) Distribution of ΔNp73α-TAP in cytoplasmic and nuclear fractions of 38HK extracts. The 2 fractions were centrifuged and analyzed by Western blotting using an anti-TAP antibody. T, total extract; S, supernatant; P, pellet. ΔNp73α-TAP is present mainly in the nuclear fraction (Cyt[P] and Nuc[S], see also Materials and Methods section). (B, lower panel) Silver-stained 10% SDS-PAGE analysis of elution fractions 1 to 5 from the second affinity purification step (calmodulin resin) for ΔNp73α-TAP and control (TAP) purifications. An additional elution with SDS was performed to recover all the remaining proteins. (C) Pathway analysis of nuclear ΔNp73α binding partners using the Reactome database (63). Only significant pathways are shown (defined by a false discovery rate [FDR] value ≤ 0.02) and ranked based on the -log_10_ of the associated *P* value (*: ≤ 0.05; **: ≤ 0.01; ***: ≤ 0.001; ****: ≤ 0.0001). Histogram bar size shows the number of input proteins involved in the corresponding pathway. See also S1 Table. (D) Sucrose gradient/co-IP experiments on endogenous 38HK proteins. (D, upper panel) Sucrose fractions of 38HK nuclear extracts stably expressing HA-ΔNp73α were migrated on a 10% SDS-PAGE gel, and analyzed by Western blotting using antibodies recognizing the HA tag, E2F4, and p130 proteins. (D, lower panel) Indicated fractions were immunoprecipitated using anti-HA conjugated beads. Unfractionated nuclear extract (NE) was loaded on the same gel as a reference for protein migration. Images for NE and sucrose fractions are derived from different exposures of the same membrane. See also original Western blot image on Mendeley data.

38HK are human keratinocytes immortalized by the E6 and E7 oncoproteins of cutaneous β-HPV38 (18). Here, we used 38HK as a cellular model to perform proteomics analyses of ΔNp73α. The entire ΔNp73α sequence was fused to the N-terminus of the tandem affinity purification (TAP) tag, cloned in a lentiviral vector under the control of the weak promoter of Moloney murine leukemia virus (22), and stably retrotransduced in 38HK (Fig. 1A). The nuclear fraction of 38HK expressing the ΔNp73α-TAP construct was recovered, and native ΔNp73α complexes purified using the TAP approach (23) (Fig. 1A and B). Finally, ΔNp73α binding partners were identified by mass spectrometry (Table S1).

10.1128/msphere.00056-23.7 TABLE S1 Nuclear protein binding partners of ΔNp73α. List of proteins displaying an enrichment of at least 10-fold in the ΔNp73α-TAP proteomics experiment compared with control (TAP-only) conditions. All proteins listed have been detected by more than 2 unique peptides, with the exception of DP1 that was detected by 2 unique peptides. Download Table S1, XLSX file, 0.01 MB. Copyright © 2023 Taverniti et al. 2023 Taverniti et al. https://creativecommons.org/licenses/by/4.0/ This content is distributed under the terms of the Creative Commons Attribution 4.0 International license.

Bioinformatics analysis of the binders show an enrichment of proteins involved in transcriptional and cell cycle control (Fig. 1C). In contrast, the E6 and E7 viral oncoproteins (38.E6 and 38.E7) do not appear to be associated with ΔNp73α.

Among the nuclear partners displaying high specificity for ΔNp73α-TAP, we identified the transcription factor E2F4 and the co-repressor retinoblastoma-like 2 (RBL2/p130) (Table S1). To corroborate this finding, we performed co-immunoprecipitation (co-IP) experiments using nuclear extracts of 38HK stably expressing ΔNp73α fused to an HA tag (HA-ΔNp73α). Extracts were first applied to a 50 to 10% sucrose gradient, and then incubated with anti-HA antibody conjugated beads. Results show that endogenous E2F4 and p130 coprecipitate with HA-ΔNp73α in fractions 11 to 12 containing all 3 proteins (Fig. 1D). By contrast, E2F4 and p130 are not recovered in fractions 13 to 16, presenting lower levels of HA-ΔNp73α, thereby confirming the co-IP specificity of factions 11 to 12. Consistently, incubation with an anti-E2F4 antibody coupled to beads coprecipitates HA-ΔNp73α and p130 in both total and fractionated 38HK nuclear extracts (Fig. S1A and B). As an alternative approach, we also fractionated 38HK nuclear extracts by size exclusion chromatography, which provides more stringent complex fractionation conditions compared to sucrose gradients. Incubation with anti-HA beads coprecipitates endogenous E2F4 and p130 proteins in fractions containing the 3 proteins (Fig. S1C).

10.1128/msphere.00056-23.1 FIG S1 Co-IP experiments on 38HK nuclear extracts. Detection of ΔNp73α, E2F4 and p130 proteins was done by Western blotting using p73 or HA tag, E2F4, and p130 antibodies, respectively. (A) Experiment on total 38HK nuclear extract using either IgG or an anti-E2F4 antibody showing co-IP of endogenous ΔNp73α and p130 proteins. Note that p130 (highlighted by a red asterix) is partially masked by a closely migrating non-specific band. (B and C) Experiments on nuclear extracts of 38HK stably expressing HA-ΔNp73α and fractionated by either sucrose gradient (B) or size exclusion chromatography (C). (Β, upper panel) Western blot analysis of sucrose gradient fractions. (B, lower panel) The indicated fractions were immunoprecipitated using anti-E2F4 antibody and analyzed for the different proteins. Unfractionated nuclear extract (NE) was loaded on the same gel as a reference for protein migration. Images for NE and sucrose fractions are derived from different exposures of the same membrane (see original Western blot image on Mendeley data). (C, upper panel) Elution profile of the 38HK nuclear extracts applied to a Superose 6 10/300 increase column. (C, middle panel) Western blot analysis of the size exclusion fractions. Fractions 20 to 30 were found to contain ΔNp73α, E2F4, and p130 proteins. (C, lower panel) The indicated fractions were immunoprecipitated using anti-HA antibody-coupled agarose beads and analyzed for the different proteins. Download FIG S1, PDF file, 0.07 MB. Copyright © 2023 Taverniti et al. 2023 Taverniti et al. https://creativecommons.org/licenses/by/4.0/ This content is distributed under the terms of the Creative Commons Attribution 4.0 International license.

These results indicate that ΔNp73α interacts with several cellular proteins, including transcriptional regulatory complexes.

### E2F4 is a binding partner of ΔNp73α.

**FIG 2:**
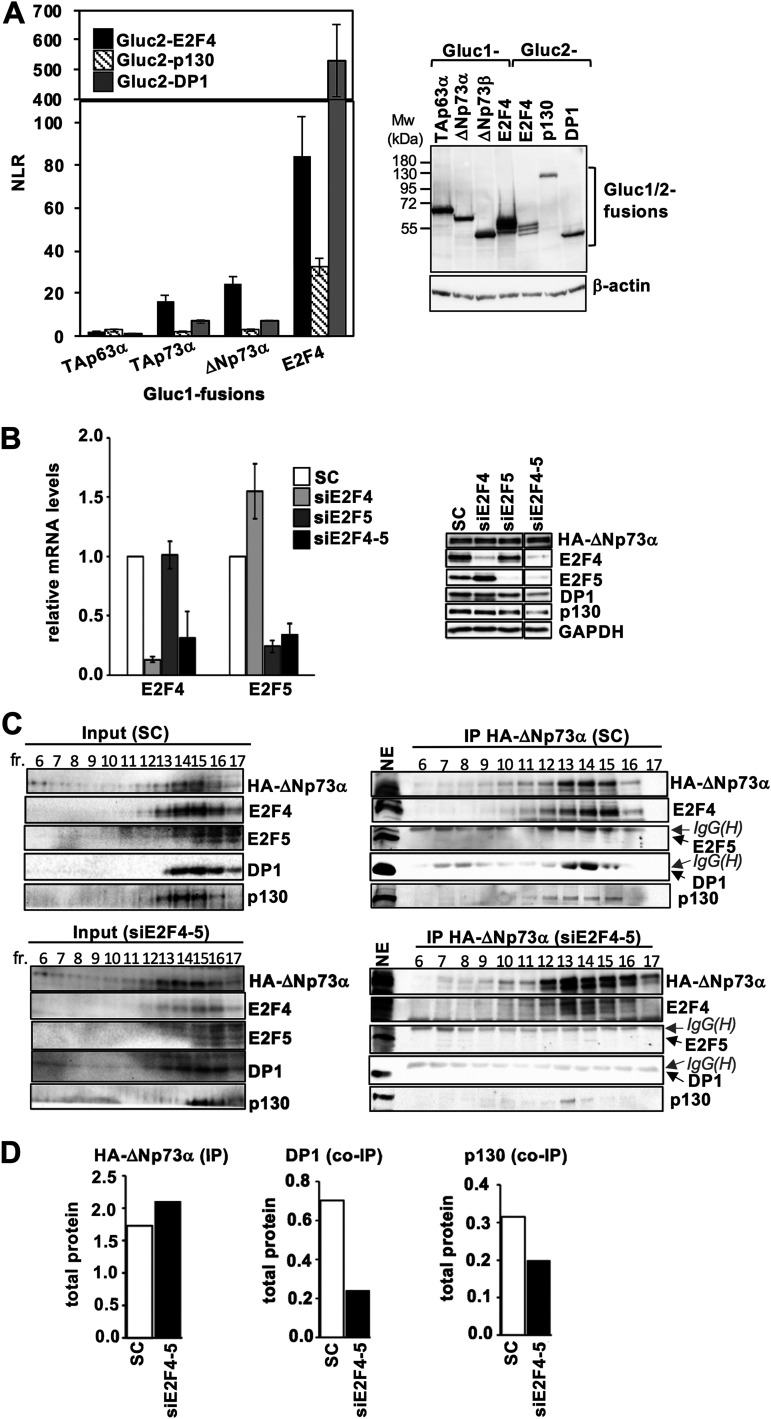
ΔNp73α establishes a direct PPI with E2F4. (A, left panel) Representative data set for GPCA analysis of binary interactions between ΔNp73/TAp63 proteins and components of the E2F4/p130 complex. Pairwise combinations of Gluc1-ΔNp73/TAp63 and Gluc2-E2F4/DP1/p130 fusion constructs were co-transfected in HEK293T cells. After 48 h, the luciferase activity was measured and expressed as normalized luminescence ratio (NLR) (27). The interactions of Gluc1-E2F4 with Gluc2-E2F4/DP1/p130 proteins are reported as internal controls. Error bars show standard deviations derived from triplicate measurements. (A, right panel) 8% SDS-PAGE analysis of the expression of Gluc1-fused ΔNp73/TAp63 and E2F4 proteins, and of Gluc2-fused E2F4, DP1 and p130 proteins in HEK293T cells. Proteins were visualized by Western blotting using an anti-Gluc antibody, which allows for detection of both Gluc1 and Gluc2 fragments, albeit with lower efficiency for Gluc2. The multiple bands detected for E2F4 likely correspond to multiple phosphorylation states of the protein as shown in (64). (B) Expression of ΔNp73α, E2F4-5, DP1, and p130 proteins in 38HK cells transfected with scramble (SC) siRNAs or siRNAs against either E2F4 (siE2F4), E2F5 (siE2F5), or E2F4 plus E2F5 (siE2F4-5). (B, left panel) mRNA levels measured by RT-qPCR. Error bars represent standard deviations derived from three independent experiments. (B, right panel) Protein levels as determined by Western blotting analysis. All samples were migrated on the same gel. See original Western blot image on Mendeley data. (C) Sucrose gradient/co-IP experiments using nuclear extracts from 38HK in the scramble (C, upper panels) and siE2F4-5 (C, lower panels) conditions. Indicated fractions were immunoprecipitated by anti-HA beads. HA-ΔNp73α, E2F4, E2F5, DP1, and p130 were detected by Western blotting. IgG(H) chains migrate in close proximity with E2F5 and DP1 proteins. NE, unfractionated nuclear extract. See also Fig. S2A and B for experiments under single siE2F4 or siE2F5 conditions. See also legend of Fig. 1D. (D) Quantification of immunoprecipitated HA-ΔNp73α DP1 and p130 proteins in the scramble and siE2F4-5 conditions of the co-IP experiments shown in panel (C). For each protein, band intensities in the individual fractions are normalized to the intensity of the corresponding band in the NE control (I_fr_/I_NE_). Then, the I_fr_/I_NE_ values of fractions 12 to 16 are summed to get an estimate of the total protein levels. The total DP1 and p130 protein values are additionally normalized to HA-ΔNp73α levels in the corresponding condition.

E2F4 forms stable heterodimers with DP1/2 (24), whereas p73 hetero-tetramerizes with p63 (25, 26). Consistently, 2 unique DP1 peptides and several p63 peptides are detected in our proteomics analyses of ΔNp73α (Table S1). To identify the binding partner of ΔNp73α, we evaluated binary interactions within a protein set that comprises ΔNp73α, TAp63α, E2F4, DP1, and p130 using the *Gaussia princeps* protein complementation assay (GPCA) (27). Here, the 2 test proteins are overexpressed in HEK293T cells as N-terminal fusions to the Gluc1 and Gluc2 inactive fragments of the *Gaussia princeps* luciferase. Based on previous benchmarking, protein pairs are considered as interacting if the normalized luminescence ratio (NLR) is above 5 (27). In our experiment, Gluc1-E2F4 and Gluc2-DP1 give a high binding response (NLR ~ 500), which is consistent with the high affinity of the E2F4/DP1 heterodimer (Fig. 2A). As a control, we also measured the interaction between Gluc1-E2F4 and Gluc2-p130. The corresponding binding response (NLR > 40) indicates that, in the conditions of overexpression of this assay, only a fraction of Gluc2-p130 is sequestered by the competing E1A and SV40 Large T antigen proteins of HEK293T cells, leaving an excess of Gluc2-p130 free for interaction with E2F4. Besides these well-established interactions, E2F4 also binds to the ΔNp73α and the ΔNp73β isoforms (NLRs > 20), but not to p130 or DP1 (Fig. 2A). Moreover, no interaction is observed for TAp63α, ruling out any direct and sequence specific contributions of this protein in complex assembly.

10.1128/msphere.00056-23.2 FIG S2 (A and B) Sucrose gradient/co-IP experiments in conditions of single E2F4 (A) or E2F5 (B) knockdown. 38HK were transfected with siRNAs against E2F4 or against E2F5. (A and B, upper panels) Sucrose fractions. (A and B, lower panels) Immunoprecipitations of sucrose fractions using an anti-HA antibody. See also legend of Fig. 1D of the main text. (C) Comparison of ΔNp73α interactions with E2F4 and E2F5. (C, left panel) Representative dataset for the GPCA analysis of Gluc1-ΔNp73α versus Gluc2-E2F4 and Gluc2-E2F5. (C, right panel) Expression levels of Gluc1-ΔNp73α, Gluc2-E2F4 and Gluc2-E2F5 in HEK293T cells. Note that the differences in binding responses between E2F5 and E2F4 are related to the different expression levels of the 2 proteins. Download FIG S2, PDF file, 0.4 MB. Copyright © 2023 Taverniti et al. 2023 Taverniti et al. https://creativecommons.org/licenses/by/4.0/ This content is distributed under the terms of the Creative Commons Attribution 4.0 International license.

Next, we evaluated protein-protein interactions (PPIs) in conditions of protein partner depletion. Knockdown of E2F4 by small interfering RNAs (siRNAs) in 38HK leads to an increase in the functional homologue E2F5, and conversely, knockdown of E2F5 raises E2F4 levels (Fig. 2B). Immunoprecipitation of HA-ΔNp73α from fractionated nuclear extracts of 38HK treated with scramble siRNA or E2F4 siRNA leads to similar recoveries of p130 and DP1 (compare Fig. 2C, upper panels with Fig. S2A). In contrast, simultaneous targeting of E2F4 and E2F5 by siRNAs strongly reduces E2F4 and E2F5 protein levels without affecting HA-ΔNp73α (Fig. 2B). In this condition, the ability of HA-ΔNp73α to coprecipitate DP1 and p130 clearly decreases (Fig. 2C, lower panels and Fig. 2D).

Together, these results show that E2F4 interacts with ΔNp73α. In conditions of E2F4 knockdown, the E2F5 homologue is upregulated and interacts with ΔNp73α.

We then compared E2F4 and E2F5 for binding to ΔNp73α by GPCA in HEK293T cells. Based on the results obtained, and taking into consideration the differences in the expression levels of the Gluc2-E2F4 and Gluc2-E2F5 fusions, we conclude that the 2 proteins are likely to interact with ΔNp73α with similar affinities under these assay conditions (Fig. S2C). However, endogenous E2F5 is not a partner of ΔNp73α, neither in the proteomics analyses (Table S1) nor in the co-IP experiments under the scramble condition (Fig. 2C, upper panels). Hence, additional factors, such as expression levels or post-translational modifications (PTMs), may account for the preference toward endogenous E2F4 in 38HK cells.

### E2F4 discriminates between TAp73 and ΔNp73 isoforms.

**FIG 3:**
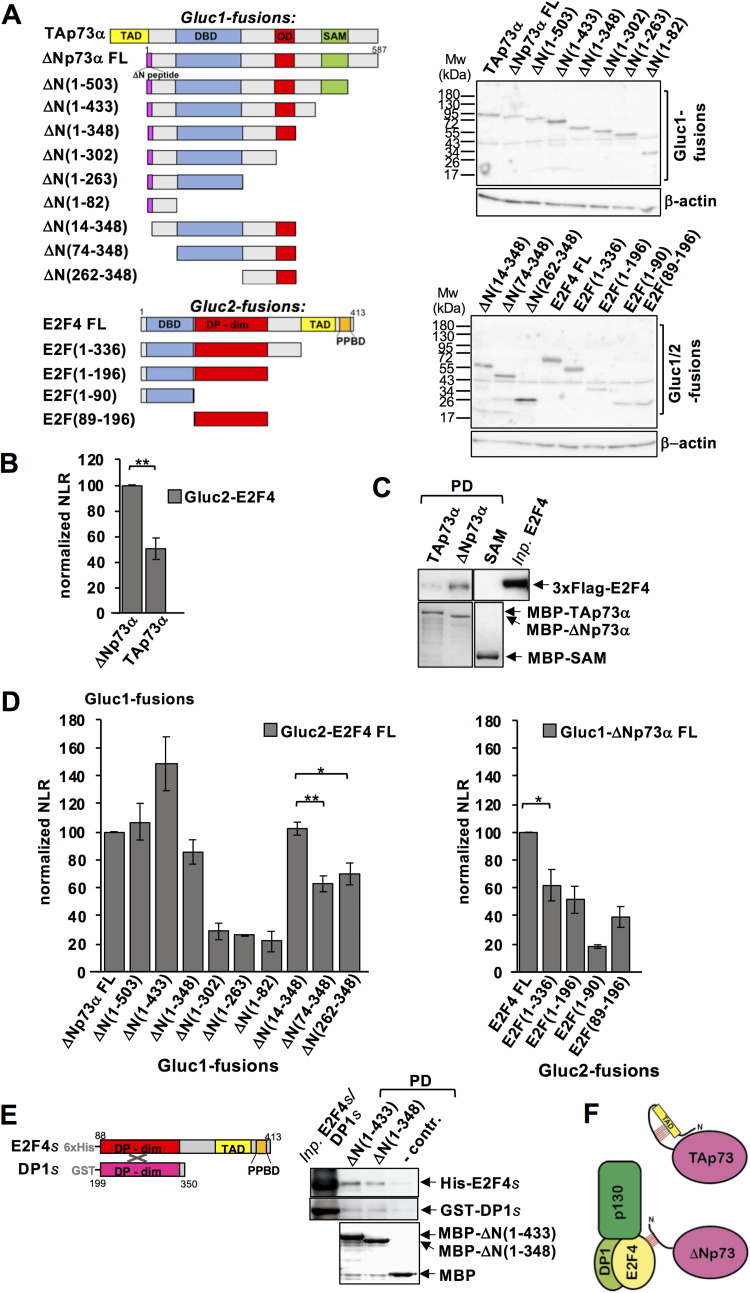
Identification of domains/regions mediating the ΔNp73α-E2F4 interaction. (A, left panel) Schematic representation of the Gluc1-fused TA/ΔNp73α and Gluc2-fused E2F4 constructs analyzed by GPCA. The ΔN(1 to 433) construct corresponds to the ΔNp73β isoform. Domains of TAp73α/ΔNp73α: transactivation domain (TAD, yellow); DNA-binding domain (DBD, blue); oligomerization domain (OD, red); sterile alpha motif domain (SAM, green); 13-amino-acid peptide specific for ΔNp73 isoforms (ΔN peptide, purple). Domains of E2F4: DNA-binding domain (DBD, blue); DP-binding and dimerization domain (DP-dim, red); transactivation domain (TAD, yellow); pocket protein binding domain (PPBD, orange). (A, right panel) Expression levels of Gluc1- and Gluc2-fused TA/ΔNp73α and E2F4 constructs in HEK293T cells. Proteins were resolved on a 25%/10% gradient SDS-gel and visualized by Western blotting using an anti-Gluc antibody. (B) GPCA analysis of the pairwise interactions of Gluc1-TAp73α or Gluc1-ΔNp73α FL with Gluc2-E2F4 FL. (C) MBP-pulldown analysis of the interactions of TAp73α and ΔNp73α FL with E2F4 FL. Amylose resin coupled to MBP-TAp73α or MBP-ΔNp73α FL constructs was incubated with clarified extracts from HEK293T cells transiently expressing 3xFlag-E2F4 FL. PD reactions were migrated on 2 separate 10% SDS-PAGE gels. One gel was used for E2F4 detection by Western blotting using an anti-Flag antibody, the second gel for detection of MBP fusions by Coomassie staining. The negative control MBP-SAM construct (residues 405 to 502 of ΔNp73α) was migrated on the same gel (see original Western blot images on Mendeley data). (D) GPCA analyses of pairwise ΔNp73α/E2F4 interactions. (D, left panel) Gluc1-ΔNp73α deletion constructs versus Gluc2-E2F4 FL. (D, right panel) Gluc1-ΔNp73α FL versus Gluc2-E2F4 deletion constructs. (B and D) The NLR values were normalized to the ΔNp73α-E2F4 FL interaction and are averages from 3 independent experiments (with each experiment being performed in triplicate). *P* values are obtained from unpaired t test, n = 3 biological triplicates (*: *P < *0.05; **: *P < *0.01). (E) Interaction analyses using recombinant purified proteins. (E, left panel) Schematic representation of the constructs used for the minimal E2F4*s*/DP1*s* heterodimer. (E, right panel) Pulldown (PD) experiment using MBP-ΔN(1 to 433) (i.e., -ΔNp73β) and MBP-ΔN(1 to 348) or MBP (negative control) proteins and the minimal E2F4*s*/DP1*s* heterodimer. PD samples were migrated on 2 separate 10% SDS-PAGE gels. One gel was used for E2F4*s*/DP1*s* detection by Western blotting using anti-His and anti GST antibodies, and the second gel for detection of MBP-ΔNp73 fusions by Coomassie staining. (F) Proposed mechanism of ΔNp73α interaction with the E2F4/p130 complex. Red hatched box: E2F4 binding site.

We reproducibly observed that the E2F4 binding response obtained with full-length TAp73α in GPCA experiments is lower (approximately 50%) than that obtained with the ΔNp73α isoform (Fig. 3A and B). This intriguing observation was confirmed by *in vitro* pulldown analyses that used recombinantly expressed full-length TAp73α and ΔNp73α proteins fused to the maltose binding protein (MBP) and clarified extracts of HEK293T cells overexpressing 3xFlag-E2F4 (Fig. 3C).

To further characterize the interaction between E2F4 and ΔNp73α, we explored the binding properties of a panel of deletion constructs of ΔNp73α and E2F4 by GPCA (Fig. 3A). First, we searched for the region of ΔNp73α that interacts with E2F4. The C-terminal half of ΔNp73α (residues 349 to 587), comprising the sterile alpha motif (SAM) domain and the disordered linker and C-terminal regions, is dispensable for E2F4 binding, thus indicating that the interaction is independent of the C-terminal splicing status (α, β, γ, δ, ε, ζ, etc.) of ΔNp73 isoforms (Fig. 3D, left panel, compare ΔNp73α FL with ΔN[1 to 348]). In contrast, deletion of the oligomerization domain (OD) strongly decreases binding, likely due to disruption of the tetrameric state and consequent loss of its avidity contributions (Fig. 3D, left panel, compare ΔNp73α FL with ΔN[1 to 302]). Hence, the minimal ΔN(1 to 348) construct, which retains the binding properties of ΔNp73α FL, was further truncated from its N-terminus. Deletion of the first 13 residues specific to ΔNp73 isoforms does not affect the interaction. In contrast, deletion of the entire N-terminal disordered region (residues 1 to 73) reduces binding to 60%, that is to levels comparable to those of full-length TAp73α, whereas further deletion of the DNA-binding domain (DBD) does not have any effect (Fig. 3D, left panel, compare TAp73, ΔNp73α FL with ΔN[14 to 348], ΔN[74 to 348], and ΔN[262 to 348]). These results indicate that the N-terminal disordered region of ΔNp73 isoforms harbors a binding site for E2F4.

Then, we searched for the region of E2F4 that interacts with ΔNp73α. Deletion of residues 197 to 413 from the disordered C-terminus of E2F4, comprising the TAD and the pocket protein binding domain (PPBD), reduces binding activity to 60% (Fig. 3D, right panel, compare E2F4 FL with E2F[1 to 336]), suggesting the existence of a binding site for ΔNp73 within this region. Further deletion of the C-terminus or of the DBD has only moderate effects on the interaction (Fig. 3D, right panel, compare E2F4 FL with constructs E2F[1 to 196] and E2F[89 to 196]), which are probably related to the lower expression levels of these constructs (Fig. 3A, right panel). In contrast, deletion of the DP-binding and dimerization (DP-dim) domain decreases binding to 20% (Fig. 3D, right panel, compare E2F4 FL with E2F[1 to 90]). This domain of E2F4 provides avidity contributions but, unlike the OD of ΔNp73α, is also a well-known region for PPIs. Therefore, a contribution of the DP-dim domain toward ΔNp73 binding cannot be excluded.

To further corroborate these findings, we performed interaction analyses using recombinant purified proteins. Since E2F4 needs to be stabilized by the cognate DP1 partner in order to be purified (24), we used co-expression approaches in bacteria to produce a minimal E2F4/DP1 heterodimer (E2F4*s*/DP1*s*), which comprises the E2F4 regions required for the interaction with ΔNp73α (i.e., DP-dim and C-terminal regions, residues 88 to 413) and the DP-dim domain of DP1 (residues 199 to 350) (Fig. 3E, left panel). In this way, we were able to obtain low concentration samples of E2F4*s*/DP1*s*, which were sufficient for pulldown analyses. Results from these analyses reproducibly show that E2F4*s*/DP1*s* interacts with 2 minimal MBP-fused ΔNp73 constructs (ΔN[1 to 433], equivalent to the ΔNp73β isoform, and ΔN[1 to 348]) but not with the MBP control (Fig. 3E, right panel).

Taken together, our results show that the minimal E2F4*s*/DP1*s* heterodimer is sufficient for the interaction with ΔNp73α. This interaction occurs through E2F4 recognition of a binding site within the N-terminal disordered region of ΔNp73 isoforms that is not accessible in TAp73, likely due to intramolecular interactions (Fig. 3F). Yet, our results do not rule out the possibility that additional protein(s) may further stabilize the ΔNp73α- E2F4 interaction *in vivo*.

### Expression and regulation of ΔNp73α-E2F4/p130 complex components in transformed versus untransformed cells.

**FIG 4:**
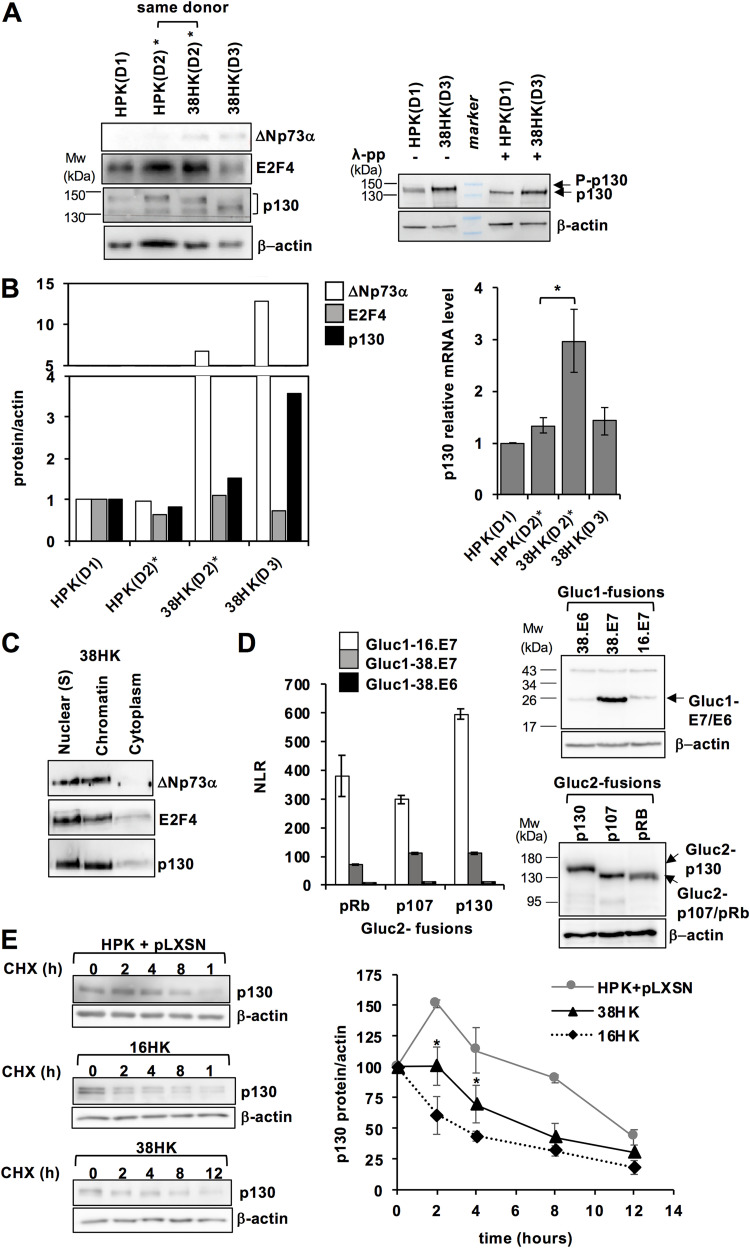
Expression and regulation of ΔNp73α-E2F4/p130 complex components in primary HPK and transformed 38HK cells. (A, left panel) Immunoblotting of ΔNp73α, E2F4, and p130 proteins in human foreskin HPK and 38HK cell lines from 3 different donors (D1 to D3). *: HPK(D2) and 38HK(D2) are from the same donor. 38HK(D2) is an early passage (i.e., passage 16) cell line, whereas the 38HK(D3) is a late passage cell line, which was used throughout this study. (A, right panel) Phosphorylation of p130. Total lysates of HPK and 38HK cells were incubated in presence or absence of λ-phosphatase (λ-pp) (8 U/μL). Note the small migration shift between the λ-pp- and λ-pp+ conditions. (B, left panel) Quantification of protein bands from the Western blot analyses shown in panel (A), left. (B, right panel) mRNA levels of the p130 gene in the different cell lines as measured by RT-qPCR. *: *P < *0.05. (C) Distribution of ΔNp73α and E2F4 and p130 proteins in the nuclear soluble (Nuclear[S]), chromatin, and cytoplasm fractions of 38HK. (D, left panel) Representative data set of the GPCA analysis of the pairwise interactions between Gluc1-38.E7/E6 or Gluc1-16.E7 proteins and Gluc2-fused RB proteins co-expressed in HEK293T cells. Error bars show standard deviations derived from triplicate measurements. (D, right panels) Expression levels of Gluc1-38.E7/E6 and Gluc1-16.E7 proteins and Gluc2-RB proteins in HEK293T cells. Gluc1-38.E7/E6 and Gluc1-16.E7 were migrated on a 12% SDS-PAGE gel, whereas Gluc2-RB proteins on a 8% gel. (E) Kinetics of p130 degradation in different keratinocyte cell lines. (E, left panels) Western blot analyses of total lysates of 38HK, 16HK, and primary HPKs stably transfected with pLXSN plasmid. All cells were treated with cycloheximide (10 μg/mL) during 12 h. Cells were collected at the indicated time points. (E, right panel) p130 levels in the three cell lines at the different time points. The data reported are normalized to actin and to the values at time = 0 (100%). Error bars represent standard deviation values derived from 3 (16HK and 38HK) or 2 (HPK + pLXSN) independent experiments. *P* values (*: *P < *0.05) refer to the differences between 38HK and 16HK at 2 and 4 h, and are calculated using the unpaired t test, n = 3 biological triplicates.

We evaluated the levels of ΔNp73α, E2F4 and p130 proteins in different primary keratinocyte (HPK) and transformed 38HK cell lines. Consistently with previous findings (18), ΔNp73α is observed in 38HK cell lines only, at both early (38HK[D2]) and late (38HK[D3]) stages of transformation (Fig. 4A and B, left panels). In contrast, the E2F4 and p130 proteins are expressed in both primary HPK and 38HKs. λ- phosphatase treatment induces a small shift in the migration of p130, which suggests that this protein is phosphorylated to a certain degree in both HPKs and 38HKs (Fig. 4A, right panel). Interestingly, the p130 protein levels appear to be higher in 38HK compared to HPK. To further investigate this observation, we measured the mRNA levels of the p130 gene by RT-qPCR. The results obtained from the cell lines from the same donor (HPK[D2] versus 38HK[D2]) show a clear increase of p130 expression upon HPV38 E6/E7 transformation (Fig. 4B, right panel).

In line with these results, analysis of the cancer database shows that E2F4, and, albeit at a lower extent, the E2F5 homologue, as well as DP1 and p130 are expressed in both normal and cancer tissues from different anatomical sites (Fig. S3). Cellular fractionation experiments show that ΔNp73α, E2F4, and p130 mostly localize to the nucleus of 38HK, and are equally distributed between the nuclear soluble (i.e., nuclear[S]) and chromatin fractions (Fig. 4C).

10.1128/msphere.00056-23.3 FIG S3 Expression level of E2F4, E2F5, TDP1, and p130 in normal and tumor tissues. The expression matrix plot of E2F4, E2F5, TDP1, and p130 in tumors (T) or normal tissues (N) has been created with the GEPIA2 tool (http://gepia2.cancer-pku.cn)- Multiple Gene Analysis-Multiple Gene Comparison. Gene list: E2F4, E2F5, TDP1, and p130. Dataset: all cancer types. Matched Normal data: TCGA tumor + TCGA normal + GTEx normal. The density of color in each block represents the median expression value of the indicated gene in a given tissue, normalized by the maximum median expression value across all blocks. Different genes in same tumors or normal tissues are compared. GTE: Genotype-Tissue Expression. ACC, Adrenocortical carcinoma; BLCA, Bladder Urothelial Carcinoma; BRCA, Breast invasive carcinoma; CESC, Cervical squamous cell carcinoma and endocervical adenocarcinoma; CHOL, Cholangio carcinoma; COAD, Colon adenocarcinoma; DLBC, Lymphoid Neoplasm Diffuse Large B-cell Lymphoma; ESCA, Esophageal carcinoma; GBM, Glioblastoma multiforme; HNSC, Head and Neck squamous cell carcinoma; KICH, Kidney Chromophobe; KIRC, Kidney renal clear cell carcinoma; KIRP, Kidney renal papillary cell carcinoma; LAML, Acute Myeloid Leukemia; LGG, Brain Lower Grade Glioma; LIHC, Liver hepatocellular carcinoma; LUAD, Lung adenocarcinoma; LUSC, Lung squamous cell carcinoma; OV, Ovarian serous cystadenocarcinoma; PAAD, Pancreatic adenocarcinoma; PCPG, Pheochromocytoma and Paraganglioma; PRAD, Prostate adenocarcinoma; READ, Rectum adenocarcinoma; SARC, Sarcoma; SKCM, Skin Cutaneous Melanoma; STAD, Stomach adenocarcinoma; TGCT, Testicular Germ Cell Tumors; THCA, Thyroid carcinoma; THYM, Thymoma; UCEC, Uterine Corpus Endometrial Carcinoma; UCS,: Uterine Carcinosarcoma. Download FIG S3, PDF file, 0.05 MB. Copyright © 2023 Taverniti et al. 2023 Taverniti et al. https://creativecommons.org/licenses/by/4.0/ This content is distributed under the terms of the Creative Commons Attribution 4.0 International license.

It is well-established that HPV E7 binds to RB family members, inhibiting their interactions with E2F factors and promoting RB protein degradation. We, thus, performed a comparative analysis of the interactions between the E7 proteins from the HPV38 and HPV16 viruses (38.E7 and 16.E7), and pRb, p107 or p130 by GPCA. Results show that, despite the high expression levels, the binding responses of 38.E7 are much lower compared to those of 16.E7 (Fig. 4D). To evaluate the impact of these different binding affinities on degradation, we assessed p130 protein levels in 38HKs versus keratinocytes transformed by the E6/E7 oncoproteins from HPV16 (16HK) or primary HPKs in conditions of cycloheximide treatment. Results show that p130 is degraded in both 16HK and 38HK, with small yet significant variations at the early time points, which point to a possible lower efficiency of the 38.E7 protein (Fig. 4E).

Together, these observations suggest that in 38HK upregulation of p130 at the transcriptional level compensates for degradation by the 38.E7 protein, thus enabling the preservation of a nuclear pool of E2F4/p130, which becomes available for interactions with other factors.

### ΔNp73α cooperates with E2F4/p130 to inhibit the expression of specific genes.

We used a three-step approach to search for genes that are targeted by both E2F4/p130 and ΔNp73α and, thus, may represent potential targets of the complex. First, we performed mRNA-seq analyses on 38HK in conditions of E2F4-5 depletion versus control. E2F4-5 knockdown by siRNAs reduces the levels of E2F4 and E2F5 proteins to 20% and 55%, respectively (Fig. S4A). This, in turn, leads to the upregulation of 2802 genes and to the downregulation of 2110 genes (adjusted *P*-value ≤ 0.05) (Fig. S5A, and Tables S2A and B). Gene set enrichment analysis of upregulated genes identifies biological processes related to cell cycle, tumor suppressor signaling, cancer, and viral infection pathways (Fig. S5B, upper panel). In contrast, downregulated genes are enriched in pathways linked to metabolism, DNA replication, and extracellular matrix-receptor interactions (Fig. S5B, lower panel).

**FIG 5:**
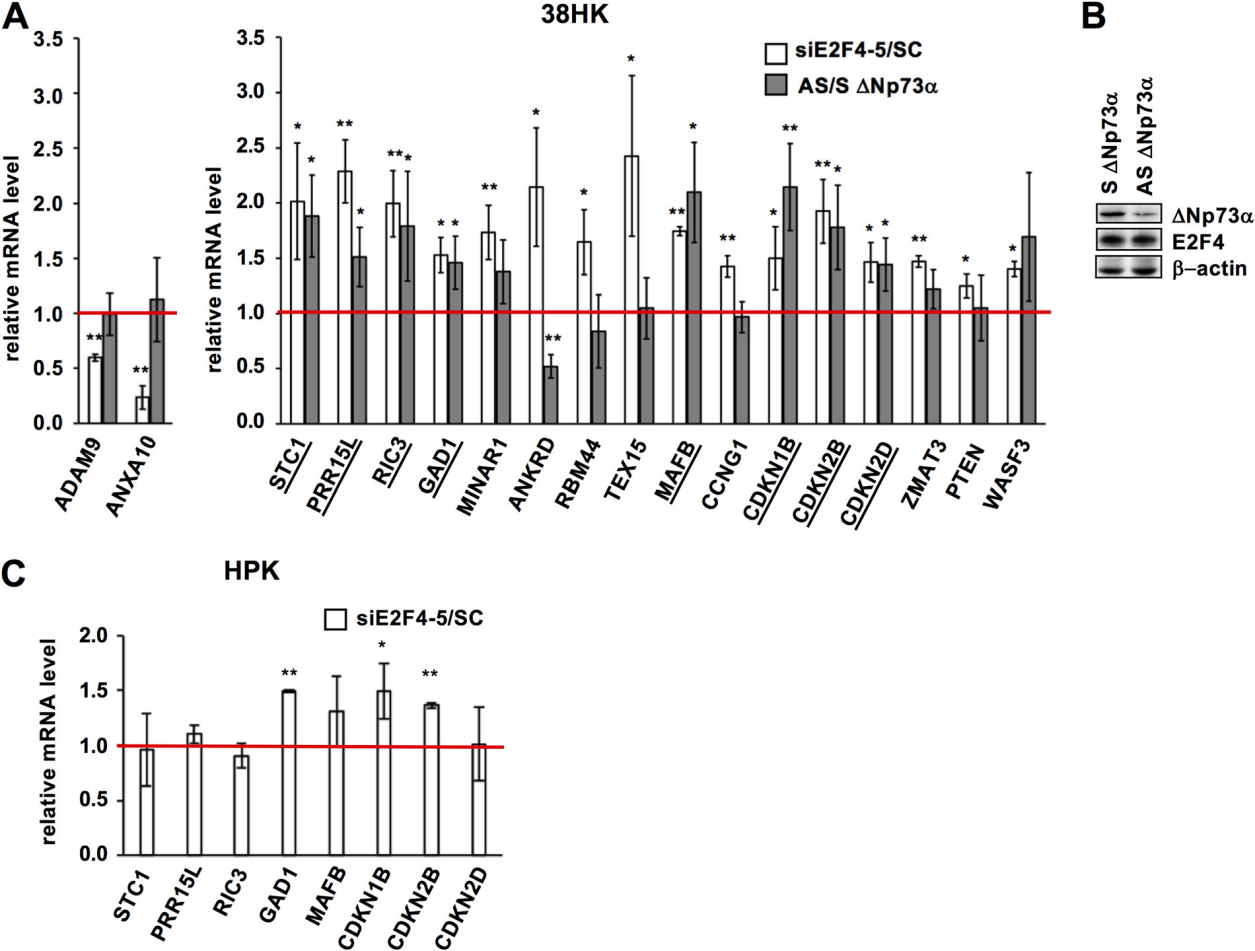
Identification of genes coregulated by E2F4-5, and ΔNp73α in 38HK. (A) Expression of selected genes in 38HK in conditions of E2F4-5 (white histograms) or ΔNp73α (gray histograms) depletion. mRNA levels are measured by RT-qPCR, first normalized to GAPDH expression, and then to the SC or S control conditions. The red line refers to the mRNA level in the SC or S conditions (set to 1). “siE2F4-5/SC” and “AS/S ΔNp73α” values > 1 and < 1 indicate upregulation and downregulation of gene expression, respectively. Underlined genes are significantly upregulated upon either E2F4 and 5 or ΔNp73α knockdown (i.e., coregulated genes). (B) ΔNp73α and E2F4 protein levels in 38HK in control and ΔNp73α knockdown conditions using S and AS oligos. See also Fig. S4B. (C) Gene expression in primary HPKs in conditions of E2F4-5 knockdown. Only genes that are coregulated by E2F4-5, and ΔNp73α in 38HK (A) are investigated. See also Fig. S4C for efficiency of knockdown in primary HPKs. (A and C) The values reported are averages of 3 independent experiments. *P* values are obtained from unpaired t test, n = 3 biological triplicates (*: *P < *0.05; **: *P < *0.01).

10.1128/msphere.00056-23.4 FIG S4 Efficiency of E2F4-5, and ΔNp73α knockdown in the different cell lines investigated in this work. Total proteins were extracted using the Nucleospin RNA/Protein kit (Macherey-Nagel). Histograms report averages that are normalized to the respective SC and S control conditions, with error bars representing standard deviation values from 3 independent experiments. (A) E2F4-5 knockdown in 38HK by siRNAs. Western blot analysis of E2F4 and E2F5 protein levels in the 38HK cultures treated with SC or siE2F4-5 RNAs, and used for mRNA-seq studies (Fig. S5). All samples were run on the same gel (see also Mendeley for original image). Protein fold changes are calculated with respect to GADPH. (B) ΔNp73α knockdown in 38HK by antisense oligos. Western blot analysis of ΔNp73α and E2F4 protein levels in 38HK cultures treated with sense (S) or antisense (AS) oligos targeting ΔNp73α. The three 38HK cultures were used for the gene expression analysis shown in Fig. 5A of the main text. Protein fold changes are calculated with respect to GADPH. (C) E2F4-5 knockdown in primary HPKs. E2F4-5 levels in HPK cultures treated with SC or siE2F4-5 RNAs. Protein fold changes are calculated with respect to β−actin. (D) E2F4-5 and ΔNp73α knockdown in cancer cells. RT-qPCR analysis of E2F4 and E2F5 expression in HNC-136 and CAL-51 cell cultures treated with SC or siE2F4-5 RNAs, and used for the gene expression analysis shown in Fig. 7B of the main text and Fig. S6. (E) Evaluation of cell death in CAL51 cultures treated with S and AS against ΔNp73α according to the optimized protocol. Total cells (adherent and floating) were colored with trypan blue. (F) ΔNp73α knockdown in cancer cells. Western blot analysis of ΔNp73α protein levels in CAL-51 cultures treated with S or AS oligos against ΔNp73α. These CAL-51 cultures were used for the gene expression analysis shown in Fig. 7B of the main text. Protein fold changes are calculated with respect to β−actin. Download FIG S4, PDF file, 0.07 MB. Copyright © 2023 Taverniti et al. 2023 Taverniti et al. https://creativecommons.org/licenses/by/4.0/ This content is distributed under the terms of the Creative Commons Attribution 4.0 International license.

10.1128/msphere.00056-23.5 FIG S5 mRNA-seq analyses in conditions of E2F4-5 knockdown versus control. (A) Volcano plot of the deregulated genes. X axis: log_2_ transformation of the fold change; genes with a positive value are upregulated, whereas genes with a negative value are downregulated. Y axis: -log_10_ transformation of the adjusted *P* value (*P*adj). Blue circles: significantly deregulated genes associated with *P*adj ≤ 0.05. Grey circles: non-significantly deregulated genes. Red circles: genes that are found to be coregulated by ΔNp73α and E2F4-5 in Fig. 5A of the main text. (B) Biological processes enriched for the genes upregulated (B, left panel) and downregulated (B, right panel) in the RNA-seq analyses. Pathway analysis was performed using the KEGG-2019 database (65). Only significant pathways are shown (defined by *P*adj ≤ 0.05) and ranked based on the associated *P* value (*: ≤ 0.05; **: ≤ 0.01; ***: ≤ 0.001; ****: ≤ 0.0001). Histogram bar size shows the number of genes involved in the corresponding pathway. Download FIG S5, PDF file, 0.2 MB. Copyright © 2023 Taverniti et al. 2023 Taverniti et al. https://creativecommons.org/licenses/by/4.0/ This content is distributed under the terms of the Creative Commons Attribution 4.0 International license.

10.1128/msphere.00056-23.8 TABLE S2 mRNA-seq analysis: genes significantly upregulated (A) or downregulated (B) upon E2F4-5 knockdown in 38HK. Significantly deregulated genes are associated with adjusted *P* values ≤ 0.05. Genes are ranked based on adjusted *P* values. The presence or absence of E2F4 and TP53/TP73 RE within the long promoter region (+2500 nt from TSS) is reported for each gene. (C) Results of E2F4 and p53/p73 RE predictions for genes upregulated upon E2F4-5 knockdown in 38HK. Sequences of upregulated genes were recovered from the Ensembl database version 86 dataset and biomaRt 2.42.0. The 2500 nt region upstream of TSS (defined as the long promoter region) was searched for E2F4 and TP53/TP73 REs using the JASPAR2020 (66) (https://github.com/da-bar/JASPAR2020) 0.99.8 and TFBSTools 1.25.2 packages. The score threshold was set to 0.85. Download Table S2, XLSX file, 0.8 MB. Copyright © 2023 Taverniti et al. 2023 Taverniti et al. https://creativecommons.org/licenses/by/4.0/ This content is distributed under the terms of the Creative Commons Attribution 4.0 International license.

In the second step, we performed RT-qPCR validation of a sample of genes from the upregulated mRNA-seq data set, with or without p53/p73 response elements (REs) within their long promoter regions (defined as 2500 nucleotides upstream of the transcription start site [TSS]) (Table S2C and Table S3). A total of 16 genes were validated with significantly higher mRNA levels in the E2F4-5 knockdown condition compared to the scramble control (Fig. 5A, right panel, white histograms, siE2F4-5/SC > 1). In this validation, we also included 2 genes from the downregulated mRNA-seq data set (ADAM9 and ANXA10), for which we confirmed inhibition upon E2F4-5 depletion (Fig. 5A, left panel, white histograms, siE2F4-5/SC < 1).

10.1128/msphere.00056-23.9 TABLE S3 Effects of E2F4-5 and ΔNp73α knockdown on the expression of selected genes in 38HK. Genes that are significantly upregulated in the RNA-seq analysis and that have been tested in classical RT-qPCR experiments. +: rescue associated with significant *P* value; -/+, rescue associated with higher average mRNA levels but not significant *P* values; -, no rescue. Download Table S3, DOCX file, 0.01 MB. Copyright © 2023 Taverniti et al. 2023 Taverniti et al. https://creativecommons.org/licenses/by/4.0/ This content is distributed under the terms of the Creative Commons Attribution 4.0 International license.

In the third step, we silenced ΔNp73α expression by treating 38HK with antisens (AS) oligonucleotides that target the N-terminal 13 aa peptide unique to ΔNp73 isoforms (Fig. 3A, left panel). Notably, knockdown of ΔNp73α induces cell death in 38HK (18) and, for this reason, a compromise between cell viability and protein depletion needs to be reached. Here, we succeeded in decreasing ΔNp73α protein levels by about 50%, while leaving E2F4 levels unchanged (Fig. 5B and Fig. S4B). ΔNp73α depletion does not affect the expression of ADAM9 and ANXA10 (Fig. 5A, left panel, gray histograms). In contrast, it enhances the mRNA levels of 8 out of the 16 upregulated and validated genes (Fig. 5A, right panel, gray histograms, AS/S ΔNp73α > 1). These genes are STC1, PRR15L, RIC3, GAD1, MAFB, CDKN1B/p27^Kip1^, CDKN2B/p15^INK4b^, and CDKN2D/p19^INK4d^. Three of these genes have no REs for p53/p73 within their long promoter regions (i.e., MAFB, GAD1, and CDKN2D/p19^INK4d^) (Table S3). Together, these results provide evidence that E2F4/p130 and ΔNp73α coregulate specific genes in 38HK.

The changes in mRNA levels observed in these experiments are limited by the only partial depletion of E2F4-5 or ΔNp73α proteins that we were able to achieve. We also attempted different protocols for triple knockdown of ΔNp73α and E2F4-5 in 38HK, which all resulted in too high toxicity, thus preventing further gene expression analyses. To evaluate the synergy between these TFs, we analyzed the expression of the 8 coregulated genes identified above albeit in primary HPKs, which do not express ΔNp73α. Results show that the expression of STC1, PRR15L, RIC3, MAFB, and CDKN2D/p19^INK4d^ is not affected by E2F4-5 silencing (Fig. 5C and Fig. S4C). In the case of CDKN2B/p15^INK4b^, the increment in expression upon E2F4-5 silencing is lower in primary HPKs compared to 38HK. This indicates that, in the absence of ΔNp73α, E2F4/p130 is unable to target these genes or inhibits their expression to a lower extent.

Hence, the synergy deriving from the ΔNp73α-E2F4 interaction can result in the re-direction of the complex to genes that are different from those targeted by the individual components.

### The ΔNp73α-E2F4 interaction enhances recruitment to DNA regulatory regions.

**FIG 6:**
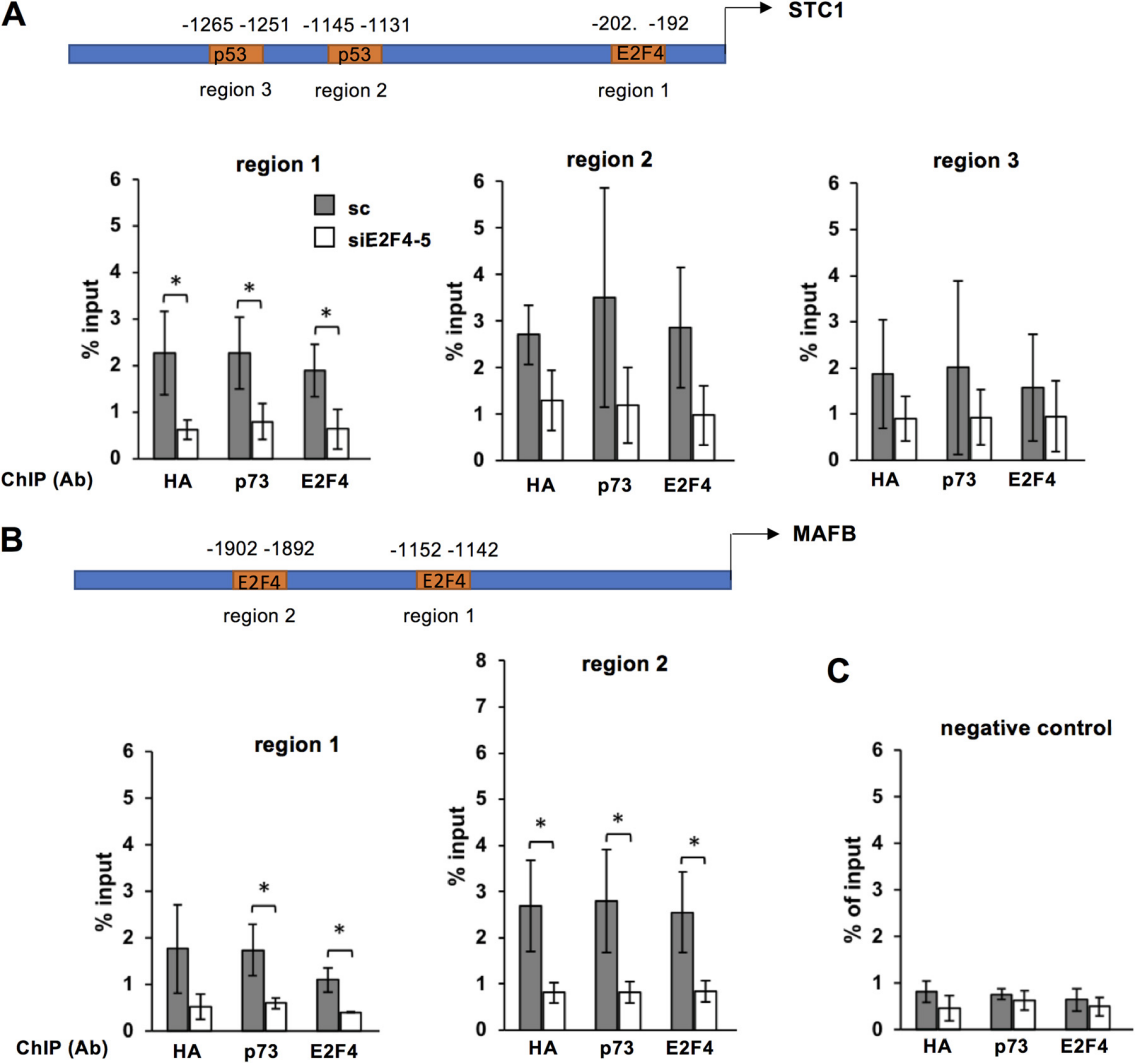
E2F4-5 knockdown decreases ΔNp73α recruitment to the promoters of the STC1 and MAFB genes. (A and B, upper panels) Schematic illustration of the long promoter regions (+2500 nucleotides from TSS) of the STC1 and MAFB genes. Numbers are the positions of TP53/TP73 and E2F4 REs associated with high prediction scores (see Table S2C). (A and B, lower panels, and C) Results from ChIP experiments. 38HK expressing HA-ΔNp73α were treated with SC (gray histograms) or siE2F4 and 5 (white histograms), and harvested at 48 h after transfection. Nuclear extracts were processed for ChIP using an anti-HA antibody recognizing HA-ΔNp73α (HA), an anti-p73 antibody recognizing both p73 and ΔNp73α proteins (p73) or anti-E2F4 (E2F4) antibodies. The eluted DNA was analyzed by qPCR with primers flanking regions 1, 2, and 3 of the STC1 promoter (A), regions 1 and 2 of the MAFB promoter (B), or the negative control region (Table S4) (C). The amount of DNA bound by each protein is expressed as percentage of DNA in the input. (A to C) The data are averages of 3 independent experiments. *P* values are obtained from unpaired t test, n = 3 biological triplicates (*: *P < *0.05).

We evaluated the recruitment of the ΔNp73α-E2F4/p130 complex to the promoter regions of coregulated genes by chromatin immunoprecipitation (ChIP). For this, we selected STC1, whose promoter hosts both E2F and p53/p73 REs, and MAFB, which hosts E2F REs only (Table S3). 38HK stably expressing HA-ΔNp73α were treated with scramble or E2F4-5 siRNAs, and processed for ChIP using anti-HA, anti-p73, and anti-E2F4 antibodies. The eluted DNA was analyzed by qPCR with primers flanking regions 1, 2, and 3 of the STC1 promoter (Fig. 6A), regions 1 and 2 of the MAFB promoter (Fig. 6B), or the negative control region reported in another transcriptomics study of E2F4 by Lee et al. (28) (Fig. 6C).

10.1128/msphere.00056-23.10 TABLE S4 Oligonucleotide sequences for gene knockdown, RT-qPCR, and ChIP experiments. Download Table S4, XLSX file, 0.01 MB. Copyright © 2023 Taverniti et al. 2023 Taverniti et al. https://creativecommons.org/licenses/by/4.0/ This content is distributed under the terms of the Creative Commons Attribution 4.0 International license.

Results show that both HA-ΔNp73 and E2F4 are recruited to regions 1 and 2 of the STC1 and MAFB genes (Fig. 6, gray histograms, compare panels A and B with panel C). E2F4-5 knockdown significantly reduces the recruitment, not only of E2F4, but also of HA-ΔNp73α to the E2F REs of region 1 of the STC1 gene, and of regions 1 and 2 of the MAFB gene (Fig. 6A and B, compare gray and white histograms). In contrast, the contributions of E2F4-5 to ΔNp73α recruitment at the p53RE of region 2 of the STC1 gene are more difficult to interpret because of the variability between the samples, which is probably due to technical limitations related to DNA shearing of this region.

These data indicate that ΔNp73α and E2F4 bind to the same promoter regions of coregulated genes. ΔNp73α recruitment to these regions takes place even in the absence of p53/p73 REs and is lost upon E2F4-5 knockdown, demonstrating its dependence on the ΔNp73α-E2F4 interaction.

### Contributions of the ΔNp73α-E2F4/p130 complex in cancer cells.

**FIG 7:**
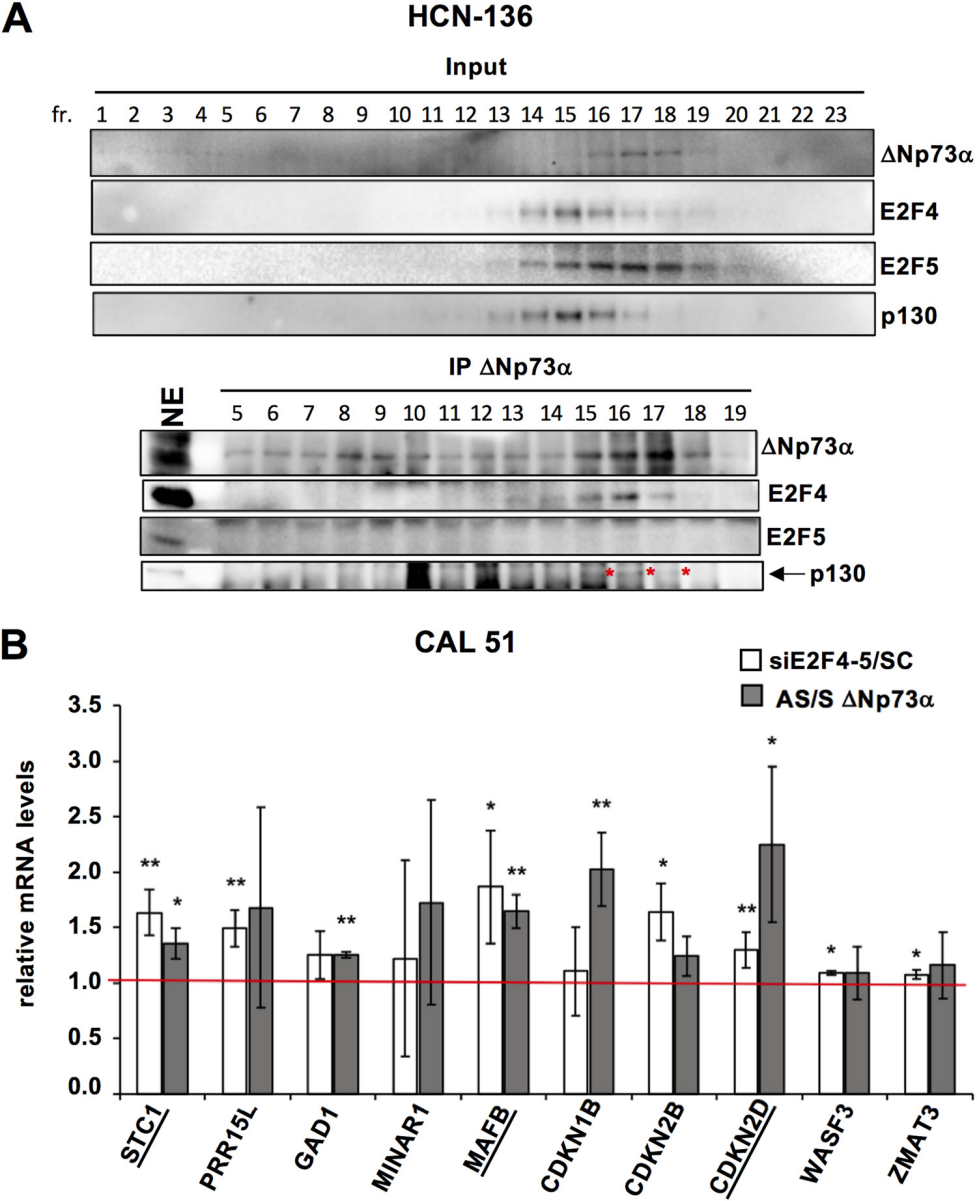
ΔNp73α cooperates with E2F4/p130 in HPV-negative cancer cells. (A) Sucrose gradient/co-IP experiments using nuclear extracts of HNC-136 cells. Sucrose fractions were immunoprecipitated using anti-p73 antibody-coupled beads and analyzed for endogenous ΔNp73α, E2F4, E2F5, and p130 proteins. Note that the p130 signal (highlighted by a red asterix) is partially masked by closely migrating nonspecific band. See also expanded Western blot images on Mendeley data. See also legend of Fig. 1D. (B) Expression profiles of selected genes in CAL-51 cells. mRNA levels determined by RT-qPCR under conditions of E2F4-5 (white histograms) or ΔNp73α (gray histograms) depletion. The red line refers to the mRNA level in the SC or S conditions (set to 1). “siE2F4-5/SC” and “AS/S ΔNp73α” values > 1 and < 1 indicate upregulation and downregulation of gene expression, respectively. The data are averages from 3 independent experiments, with error bars representing standard deviation values. *P* values are obtained from unpaired t test, n = 3 biological triplicates (*: *P < *0.05; **: *P < *0.01; ***: *P < *0.001). See also Fig. S4D to F for E2F4-5, and ΔNp73α levels in the scramble and knockdown conditions.

Because several studies have highlighted the importance of ΔNp73α in different types of cancer, we evaluated whether the ΔNp73α-E2F4 interaction occurs in cancer-derived cell lines. We previously identified an HPV-negative head and neck cancer cell line (HNC-136) and a breast cancer cell line (CAL-51), both with high levels of ΔNp73α and wild-type TP53 (20). Nuclear extracts from HNC-136 were treated by sucrose density gradient. Immunoprecipitation of endogenous ΔNp73α using anti-p73 antibody-coupled beads leads to recovery of endogenous E2F4 and p130 proteins (Fig. 7A, lower panel, fractions 15 to 17), thereby confirming ΔNp73α-E2F4/p130 complex formation in these cells. Interestingly, the complex from HNC-136 extracts migrates in fractions of lower sucrose density compared to the complex from 38HK extracts (compare Fig. 7A with Fig. 1D). This suggests that ΔNp73α-E2F4/p130 may include auxiliary proteins that depend on the cellular context. In addition, although HNC-136 extracts exhibit clear expression of E2F5, no E2F5 is recovered upon ΔNp73α immunoprecipitation.

Then, we evaluated the expression levels of the genes, which are coregulated by ΔNp73α and E2F4/p130 in 38HK. E2F4-5 depletion in both HCN-136 and CAL-51 (Fig. S4D) increases the mRNA levels of most of these genes (Fig. 7B, white histograms, and Fig. S6). In contrast, ΔNp73α silencing by antisens oligonucleotides induces cell death at levels that are even higher than those observed for 38HK. However, by carefully screening oligonucleotide concentration, we were able to identify one condition for the CAL-51 cell line, which limits the cell death (Fig. S4E) and results in a ΔNp73α knockdown to approximately 60% at 24 h after transfection (Fig. S4F). Remarkably, even with such a partial depletion, we can observe an increase in the expression of several genes, including 3 genes, STC1, MAFB, and CDKN2D/p19^INK4d^, which are also downregulated by E2F4/p130 (Fig. 7B, gray histograms). As described in the previous section, these latter 3 genes are unaffected by E2F4-5 depletion in primary HPKs lacking ΔNp73α (Fig. 5C).

10.1128/msphere.00056-23.6 FIG S6 Expression profiles of selected genes in HNC-136 cells. mRNA levels of selected genes determined by RT-qPCR in conditions of E2F4-5 knockdown. The red bar corresponds to the expression level of each gene in the scramble or sense control conditions (set to 1). “E2F4-5 siRNA/scramble” values > 1 and < 1 indicate upregulation and downregulation of gene expression, respectively. Error bars report on the standard deviations from 3 independent experiments. *: *P < *0.05; **: *P < *0.01. See also legend of Fig. 5 of the main text. Download FIG S6, PDF file, 0.04 MB. Copyright © 2023 Taverniti et al. 2023 Taverniti et al. https://creativecommons.org/licenses/by/4.0/ This content is distributed under the terms of the Creative Commons Attribution 4.0 International license.

Together, our results suggest that the ΔNp73α-E2F4/p130 complex regulates the expression of a core set of specific genes in different transformed cell lines.

### p19^INK4d^ expression induces senescence in 38HK.

**FIG 8:**
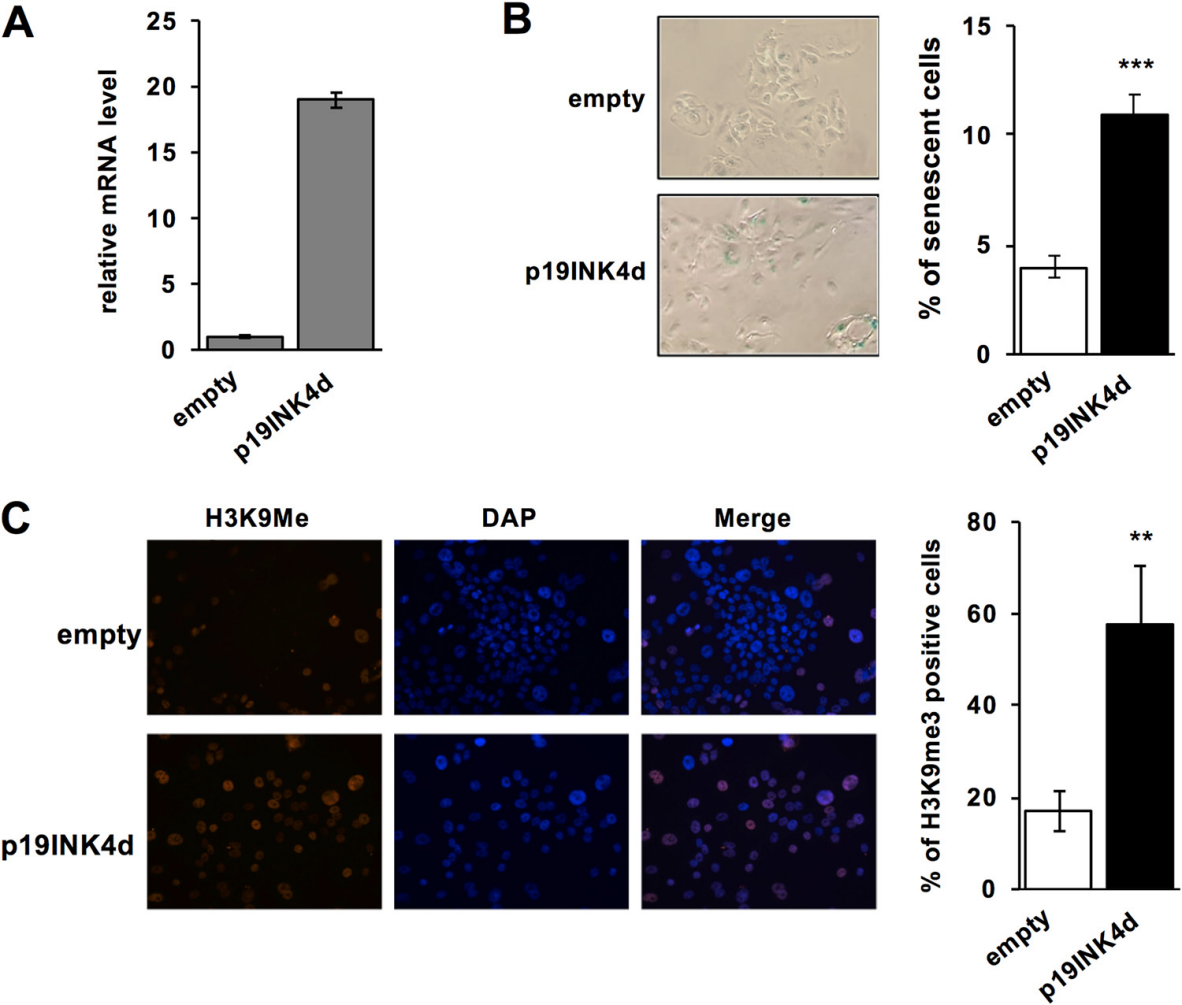
Expression of p19^INK4d^ induces senescence in 38HK. (A) p19^INK4d^ mRNA levels determined by RT-qPCR in 38HK retro-transduced with pMSCV-p19^INK4d^-IRES-GFP or with pLXSN-GFP (negative control). (B) *In situ* senescence-associated β-galactosidase staining. (B, left panel) Representative photomicrographs of β-galactosidase staining at pH 6. (B, right panel) Percentage of senescent 38HK in the negative control and p19^INK4d^ expression conditions. The data are averages and standard deviations from 3 independent experiments. (C) Immunofluorescent staining for SAHF. (C, left panel) Representative immunofluorescence images. Cells were layered on coverslips coated with polylysine and probed using an anti-H3K9me3 antibody, followed by secondary Alexa Fluor 555-conjugated antibody. Nuclei were stained with DAPI (colored blue). (C, right panel) Quantification of cells positive for H3K9me^3^ marks in the in the negative control and p19^INK4d^ expression conditions was performed using ImageJ software. (A to C) The data are averages from 3 independent experiments, with error bars representing standard deviation values. (B to C) More than 100 cells were counted for each condition. *P* values are obtained from unpaired t test, n = 3 biological triplicates (**: *P < *0.01, ***: *P < *0.001).

The CDKN2D/p19^INK4d^ gene is repressed by ΔNp73α, and E2F4-5 in both 38HK and CAL51 cancer cell lines. A previous study showed that the p19^INK4d^ protein is involved in senescence by contributing to formation of senescence-associated heterochromatin *foci* (SAHF) (29). To test whether similar mechanisms occur in our model system, we retro-transduced p19^INK4d^ or negative control pLXSN-GFP plasmid in 38HK (Fig. 8A), and performed senescence analyses. Results show that expression of p19^INK4d^ induces a 2-fold increase in the number of senescent cells in the β-galactosidase assay (Fig. 8B) and a 3-fold increase in the number of cells positive for the H3K9me3 marker of SAHF *foci* (Fig. 8C). This suggests that repression of the CDKN2D/p19^INK4d^ gene by the ΔNp73α-E2F4/p130 complex favors cell survival.

## DISCUSSION

In this study, we present what is, to the best of our knowledge, the first reported proteomics analysis on a ΔNp73 isoform, which has enabled us to identify the E2F4/p130 repressor complex as a nuclear partner of ΔNp73α. E2F4 belongs to the E2F family of proteins, whose members act as transcriptional activators (E2F1/2/3/6) and repressors (E2F4-5). E2F TFs associate with DP1–4 proteins through the dimerization domain and interact with the retinoblastoma (RB) family proteins (pRb, p130, p107) via the TAD. This interaction with RB proteins prevents recruitment of the transcriptional machinery. In the canonical model of cell cycle progression, E2F4 and E2F5 mediate the repression of cell cycle genes and cell cycle arrest in the G0 phase and are part, together with p107/p130, DP1/2, and MuvB, of the DREAM complex (30, 31). Here, besides E2F4, DP1, and p130, none of the other DREAM complex subunits (i.e., LIN proteins, RBBP4) could be detected in association with ΔNp73α, suggesting that ΔNp73α-E2F4/p130 and DREAM are distinct complexes. In addition to the canonical functions, E2F4 has also activator functions that are RB independent (32). Here, E2F4-5 depletion in 38HK leads to downregulation of a large set of genes related to different biological pathways.

E2F4-5 and associated proteins (DP1 and p130) are expressed in both healthy and cancer tissue. In this work, we unveil a direct PPI between E2F4 and ΔNp73α. Despite the homology with E2F5, E2F4 appears to be the preferred partner of ΔNp73α in transformed cells. This is in line with previous studies showing oncogenic functions for E2F4 but not for E2F5 (33–35).

Many of our analyses are performed in the 38HK model of viral transformation, in which ΔNp73α is the main expressed isoform, whereas TAp73 levels are under the detection limit (18). Neither the E6 nor the E7 viral proteins were detected among the ΔNp73α partners. Interestingly, we have also observed an increase in the expression of the p130 gene in 38HK compared to primary HPKs, which appears to compensate for the degradation of the p130 protein by 38.E7. Furthermore, the detection of a ΔNp73α-E2F4/p130 complex in extracts of the HPV-negative HNC-136 cancer cell line confirms that the ΔNp73α-E2F4 interaction is independent of the viral oncoproteins.

Results from binding analyses suggest that truncation of the TAD region of p73, giving rise to ΔN isoforms, leads to the exposure of a binding site for E2F4 and that this interaction mechanism is independent of the C-terminal splicing status of ΔNp73 isoforms. This, in turn, also suggests that the interactome of ΔNp73 isoforms may be quite different from that of TAp73. This situation is reminiscent of the gain-of-function PPIs established by oncogenic mutants of p53 with other TFs (36, 37). However, unlike mutant p53 interactions, which generally lead to activation of transcription, the interaction of ΔNp73α with the E2F4/p130 complex results in the inhibition of gene expression.

It is well established that direct PPIs between TFs belonging to different families enhance cooperative binding to DNA and that this can result in TF redirection to different sites (38–40). Here, we show that most genes, which are found to be coregulated by ΔNp73α and E2F4/p130 in 38HK, are not targeted by E2F4/p130 in primary HPKs lacking ΔNp73α. Then, in complementary ChIP experiments, we demonstrate that E2F4 mediates recruitment of ΔNp73α to E2F REs. These findings provide evidence that the ΔNp73α-E2F4 interaction modulates the DNA-binding specificities of both ΔNp73α and E2F4/p130.

In this work, we have identified 3 genes, namely, STC1, MAFB, and CDKN2D/p19^INK4d^, that are repressed by either ΔNp73α or E2F4/p130 in 2 different transformed cell lines (38HK and CAL51), and which are not targeted by E2F4/p130 in primary HPKs lacking ΔNp73α. Stanniocalcin 1, the product of the STC1 gene, is one of the most highly secreted factors in senescent cells (41). MAFB instead belongs to the AP1 family of TFs (42). Besides its well-established role as an oncogene, MAFB acts as a tumor suppressor in certain cellular contexts, by antagonizing oncoproteins, such as HRAS and BRAF (43). CDKN2D/p19^INK4d^ belongs to the *Cdkn* family, which encodes for negative regulators of cell cycle progression by binding and inhibiting cyclin-dependent kinases CDK1/2 (p27^Kip1^) and CDK4/6 (p27^Kip1^, p15^INK4b^, and p19^INK4d^) (44–46). p19^INK4d^ has been shown to have antiproliferative properties and to be inactivated in several human cancers (47–49). In agreement with a previous study (29), here we find that expression of p19^INK4d^ induces cellular senescence associated to heterochromatin formation in 38HK.

Altogether, our results on the repression of specific genes implicated in cellular proliferation/survival suggest that the interaction with oncogenic ΔNp73α may alter the biological properties of E2F4 in transformed cells. Yet, our data do not exclude that ΔNp73α or E2F4/p130 act alone or as part of other complexes also exerting oncogenic functions.

Our interaction analyses suggest that the E2F4 binding site is hindered in TAp73 by intramolecular interactions. p73 proteins are known to encompass a C-terminal transcription inhibitory domain (TID), which transiently interacts with the TAD domain (50). Deletion of the TID domain did not increase TAp73 binding to E2F4 (data not shown), suggesting that TID is not involved in the masking of the E2F4 interaction site. Future biochemical and structural studies are required to understand the precise mechanisms underlying the ΔNp73α-E2F4 interaction.

In conclusion, our study shows that, in transformed cells, the ΔNp73α isoform interacts with and redirects the E2F4/p130 transcriptional complex to specific genes.

## MATERIALS AND METHODS

### DNA constructs.

The ORFs encoding human E2F4, human E2F5, and human DP1 were purchased from Addgene, whereas the ORF encoding human p130 from Origene.

**(i) Retroviral constructs.** ΔNp73α-TAP was generated in 2 steps: First, the ΔNp73α open reading frame (ORF) was amplified by PCR and cloned in the pCDNA-TAP vector (23). Second, the pCDNA ΔNp73α-TAP plasmid was used as a template for PCR amplification of the ΔNp73α-TAP fusion, which was cloned into pBABE-puro retroviral vector (22) (pBABE ΔNp73α-TAP). For HA-ΔNp73α, the ΔNp73α ORF was amplified with an oligo complementary to its N-terminus and containing the HA tag and directly cloned into pBABE-puro (pBABE HA-ΔNp73α). pMSCV-p19Ink4d-IRES-GFP plasmid expressing mouse p19INK4d (sharing 87% sequence identity with human p19INK4d) (51) was obtained from Addgene.

**(ii) Constructs for expression in mammalian cells.** ORFs encoding for ΔNp73α, TAp73α, TAp63α, E2F4, E2F5, DP1, and p130 were amplified by PCR, and cloned into the pDONR207 vector by recombination cloning (Gateway system, Invitrogen). The resulting pEntry clones were then transferred into the GPCA destination vectors pSPICA-N1 and pSPICA-N2 (27), and into a pcDNA3 vector enabling expression of an N-terminal 3xFlag tag.

**(iii) Constructs for Escherichia coli expression.** ORFs encoding for ΔNp73α, TAp73α, and constructs of these proteins were cloned in the NcoI and KpnI sites of a modified pETM-41 vector containing an N-terminal MBP tag followed by a TEV cleavage site. The E2F4(84 to 413) and DP1(199 to 350) DNA constructs for expression of the minimal E2F4*s*/DP1*s* heterodimer were cloned in the NdeI and BamHI sites of the pmCS and pnEA vectors, which allow for fusion to N-terminal 6×His and GST tags, respectively (52).

All constructs were verified by DNA sequencing.

### Cell lines and cell culture.

Phoenix, NIH 3T3, HEK293T, HNC-136, and CAL-51 cells were cultured in Dulbecco’s modified Eagle’s medium (DMEM), supplemented with 10% calf serum (NIH 3T3) at 37°C with 5% CO_2_.

38HK (53) were grown together with NIH 3T3 feeder layers in FAD medium containing 3 parts Ham’s F12, 1 part DMEM, 2,5% fetal calf serum, insulin (5 μg/mL), epidermal growth factor (10 ng/mL), cholera toxin (8.4 ng/mL), adenine (24 μg/mL), and hydrocortisone (0.4 μg/mL). Feeder layers were prepared by treating NIH 3T3 with mitomycin C for 2 h. Around 3x10^5^ of treated NIH 3T3 were cocultured with 38HK cells in T75 cell culture flasks. Feeder layers were removed by incubating the cell cocultures with 5 mL of PBS 1X supplemented with 2 mM EDTA. In this way, more than 95% of the feeder cells were removed. Then, 38HK cells were collected by scraping for analysis.

HPK cells were freshly isolated from neonatal foreskin and cultured in Keratinocyte Growth Medium 2 (PromoCell).

### Cell line generation.

Retrovirus transduction system was used to generate 38HK cells stably expressing ΔNp73α-TAP, HA-ΔNp73α, and p19INK4d constructs. High-titer retroviral supernatants were generated by transient transfection of Phoenix cells with the retroviral constructs described above and used to infect 38HK, as described previously (54). Briefly, 500 μL of DNA mix (10 μg plasmid DNA, 248 mM CaCl_2_) were gently mixed to 500 μL of 2X HBS-buffered saline (1.5 mM Na2HPO4, 50 mM HEPES, 280 mM NaCl, 10 mM KCl, 12 mM Dextrose, and pH 7.05). The transfection mix was then used to transfect Phoenix cells cultured in 5 mL fresh medium supplemented with 25 μM Chloroquine for 6 to 8 h. After 48 h, the culture medium containing the retrovirus was filtered (0.2 μm filter), mixed with 5 μL of Polybrene (Sigma), and used to infect 38HK cell cultures for 3 h. After 24 h of infection, 38HK were selected in 0.2 μg/mL of puromycin for 3 to 5 days.

### Transfection conditions.

38HK, HNC-136, and CAL-51 cell lines were transiently transfected with siRNAs and sense/antisense oligonucleotides (Table S4) using Lipofectamine 2000 (Invitrogen). After 4 h, the transfection mix was removed, and the cells cultured in FAD medium (without antibiotic and cholera toxin). HEK293T cells were transfected using JetPEI (Polyplus transfection).

### Proteomics.

38HK (about 2x10^8^ total cells) stably expressing ΔNp73α-TAP or TAP tag alone (negative control) were resuspended in 8 mL of cold buffer A (10 mM HEPES-KOH pH 7.9, 1.5 mM MgCl_2_, 10 mM KCl, 0.5 mM DTT, 0.2 mM EDTA, protease inhibitor cocktail mix 1x, and 10 mM NaF) and incubated for 30 min on ice. Then, cells were lysed by passing the mix through a 25-gauge needle 15 times, and centrifuged for 10 min at 13400 rpm at 4°C. The supernatant (cytoplasmic soluble fraction) was flash-frozen and stored at −80°C, while the pellet (the nuclear fraction) was resuspended in 7 mL of cold buffer B (20 mM HEPES-KOH pH 7.9, 1.5 mM MgCl_2_, 250 mM NaCl, 20% glycerol, 0.5 mM DTT, 0.2 mM EDTA, protease inhibitor cocktail mix 1x, and 10 mM NaF) and incubated on ice for 1 h. The mix was centrifuged for 10 min at 13,400 rpm at 4°C and the supernatant (nuclear soluble fraction) was collected. Each lysis step was checked by Western blotting (Fig. 1B, upper panel).

Buffer A was added to the nuclear soluble fraction to reach a final concentration of 200 mM NaCl and 16% glycerol. The resulting nuclear protein extract was transferred to an ultra-clear polycarbonate tube and centrifuged at 40,000 rpm for 1 h at 4°C using a SW41 rotor (Beckmann). After centrifugation, the supernatant was carefully collected and incubated overnight with 100 μL (dry bead volume) of prewashed IgG Sepharose beads. Beads were then washed 3 times with 10 mL of IPP150 buffer (10 mM Tris-Cl pH 8.0, 150 mM NaCl, and 0.1% NP40) and once with 10 mL of TEV buffer (10 mM Tris-Cl pH 8.0, 150 mM NaCl, 0.1% NP40, 0.5 mM EDTA, and 1 mM DTT). Subsequently, beads were resuspended in 1 mL of TEV cleavage buffer containing 15 μL (10 U/μL) of acTEV protease (Invitrogen) and incubated with gentle agitation for 4 h at 16°C. Protein complexes were eluted by gravity flow. Then, 3 mL of calmodulin binding buffer (10 mM β-mercaptoethanol, 10 mM Tris-Cl pH 8.0, 150 mM NaCl, 1 mM magnesium acetate, 1 mM imidazole, 2 mM CaCl_2_, and 0.1% NP40) and 3 μL of 1 M CaCl_2_ were added to the 1 mL eluate containing the protein complexes to chelate the EDTA present in the TEV cleavage buffer. The resulting mix was incubated with 100 μL of prewashed (dry bead volume) calmodulin-Sepharose beads (Agilent Technologies) for 2 h with gentle agitation at 4°C. Beads were then washed 3 times with 10 mL of calmodulin binding buffer. The protein complexes were recovered with 5 consecutive elutions (200 μL each) with calmodulin elution buffer (10 mM β-mercaptoethanol, 10 mM Tris-Cl pH 8.0, 150 mM NaCl, 1 mM magnesium acetate, 1 mM imidazole, 2 mM EGTA, and 0.1% NP40). An additional elution with 1% SDS was performed to recover all the remaining proteins (Fig. 1B, lower panel).

Elution fractions 2 from the ΔNp73α-TAP and TAP purifications were partially digested with trypsin and analyzed by LC-MS using an Orbitrap ELITE instrument equipped with a C18 Accucore 50 cm column. The generated data were analyzed using the Proteome Discoverer 2.4 tool. Proteins enriched more than 10-fold in ΔNp73α-TAP compared with the control (TAP-only) experiment are listed in Table S1. Enrichment is calculated from the ratio of the sums of peptide peak areas in test and control experiments.

### Cellular fractionation.

38HK (out 2.5x10^7^ total cells) stably expressing HA-ΔNp73α were resuspended in 1 mL of cold buffer A (described in the proteomics section) and incubated for 15 min on ice. Then, cells were lysed by passing the mix through a 25-gauge needle 15 times and centrifuged for 5 min at 12000 rpm at 4°C. The supernatant, corresponding to the cytoplasmic soluble fraction, was recovered, while the pellet (the nuclear fraction) was resuspended in 200 μL of cold buffer B (described in the proteomics section), and incubated on ice for 30 min. Then, the mix was passed through a 27-gauge needle 15 times and centrifuged for 10 min at 12000 rpm at 4°C. The supernatant (i.e., the nuclear extract) was recovered and used for the co-IP experiments.

### Sucrose gradient/co-IP.

Sucrose density gradients were performed as previously described (55), with minor modifications. Briefly, step gradients were made by superposing sucrose solutions of different concentration (50%, 40%, 30%, 20%, and 10%) in an ultra-clear polycarbonate tube (Beckman), and a linear gradient was allowed to form overnight at 4°C. Then, 1.5 to 2 mg of nuclear extracts from 38HK HA-ΔNp73α or HNC-136 cells were carefully transferred to the top of the sucrose gradient and the protein complexes were separated based on their molecular weight by ultracentrifugation at 35,300 rpm for 16 h at 4°C using the SW41 rotor. After centrifugation, 500 μL fractions were collected from the bottom of the tube by gravity flow.

ΔNp73α complexes were immunoprecipitated from each fraction using 30 μL of slurry anti-HA-agarose beads (Sigma-Aldrich, ref. A2095) or 20 μL of pre-coupled anti-E2F4 or anti-p73 Sepharose beads (dry bead volume). Briefly, each fraction was incubated with the beads for 2 h (HA beads) or 4 h (E2F4 and p73-beads). After incubation, the beads were washed 5 times with 1 mL of washing buffer (20 mM Tris-HCl pH 7.5, 1.5 mM MgCl_2_, 150 mM NaCl, 0.2 mM EDTA, and 0.1% Igepal). The protein complexes were eluted in 1x loading dye buffer.

### GPCA assay.

HEK293T cells were transfected using the reverse transfection method. Transfection mixes containing 100 ng of pSPICA-N2 and 100 ng of pSPICA-N1 plasmids expressing test protein, plus JetPEI (Polyplus transfection) were dispensed in white 96-well plates. HEK293T cells were then seeded on the DNA mixes at a concentration of 4.2 x10^4^ cells per well. At 48 h after transfection, cells were washed with 50 μL of PBS and lysed with 40 μL of Renilla lysis buffer (Promega, E2820) for 30 min with agitation. *Gaussia princeps* luciferase enzymatic activity was measured using a Berthold Centro LB960 luminometer by injecting 50 μL per well of luciferase substrate reagent (Promega, E2820) and counting luminescence for 10 s. Results are expressed as a fold change normalized over the sum of controls, specified herein as normalized luminescence ratio (NLR) (27). For a given protein pair A/B, NLR = (Gluc1-A + Gluc2-B)/[(Gluc1-A + Gluc2) +(Gluc1 + Gluc2-B)].

### Protein expression in E. coli and pulldown assays.

The minimal E2F4*s*/DP1*s* heterodimer was produced by co-expression of 6×His-E2F4 (84 to 413) and GST-DP1(199-350) in E. coli BL21 DE3 cells overnight at 15°C. The bacterial pellet (500 mL expression) was resuspended in lysis buffer (20 mM Tris pH 8.0, 400 mM NaCl, 10% glycerol, 5 mM DTT, lysozyme, 100 μg/mL DNase I, 100 μg/mL RNase, and cOmplete EDTA-free [Roche]) and lysed by sonication. Cleared extracts were applied to Ni^2+−^NTA resin previously equilibrated in buffer A (20 mM Tris pH 8.0, 400 mM NaCl, 10% glycerol, and 2 mM DTT). After extensive washing, the E2F4/DP1 heterodimer was eluted by applying buffer A supplemented with 250 mM imidazole. Subsequently, the sample was concentrated and then buffer exchanged using a Nap10 (GE health care) column equilibrated in buffer B (20 mM Tris pH 8.0, 150 mM NaCl, 10% glycerol, and 2 mM DTT).

Over-expression of ΔNp73α and TAp73α proteins (full-length and truncated constructs) fused to MBP was carried out overnight in E. coli BL21 DE3 cells at 15°C. Cell pellets (50 mL expressions) were resuspended in lysis buffer (20 mM Tris pH 8.0, 250 mM NaCl, 10% glycerol, 0.2% NP-40, 2 mM DTT, lysozyme, 100 μg/mL DNase, 100 μg/mL RNase, and cOmplete EDTA-free [Roche]), lysed by sonication, and cleared by centrifugation. Supernatants were then incubated with 100 μL of pre-equilibrated amylose resin beads for 2 h at 4°C. Subsequently, resin was extensively washed with PD buffer (20 mM Tris pH 8.0, 150 mM NaCl, 2 mM DTT, and cOmplete EDTA-free). For the pulldown experiment, 10 μL of amylose resin coupled to MBP-p73/ΔNp73α proteins were incubated with clarified lysates of HEK293T expressing 3xFlag-E2F4 or recombinant purified E2F4*s*/DP1*s* heterodimer for 2 h at 4°C. After 2 quick washing steps with PD buffer, complexes were eluted by incubation with 20 μL of PD buffer supplemented with 20 mM maltose for 15 min at 4°C. PD reactions were migrated onto 2 separate 10% SDS-PAGE gel. One gel was used for Western blot to detect E2F4 or E2F4*s*/DP1*s*, the other for Coomassie staining to detect MBP-p73/ΔNp73α proteins.

### Immunoblotting.

Western blot detection of endogenous proteins was performed using the following antibodies: p73 (Abcam, ref: ab215038), E2F4 (Santa Cruz Biotechnology, ref. sc-398543X), E2F5 (Genetex, ref. GTX129491), DP1 (Abcam, ref. ab124678), p130 (Cell Signaling, ref. 13610S) β-actin (clone C4, MP Biomedicals), and GAPDH (6C5, ref. sc-32233, Santa Cruz). Detection of tagged constructs was done using: HA-peroxidase antibody (Roche, ref: 12013819001), anti-TAP antibody (Thermofisher Scientific, ref. CAB1001), anti-Flag antibody (Sigma, ref. F3165) antibody, and anti-Gluc antibody (New England Biolabs, ref. E8023).

### mRNA-seq.

38HK cells transfected with scramble siRNA or E2F4-5 siRNAs were collected at 48 h after transfection. Total RNA was extracted from 38HK (about 10^6^ cells per sample) using the RNeasy minikit from Qiagen and quantified by Qubit.

A total of 6 samples (3 for scramble siRNA and 3 for E2F4-5 siRNA) were analyzed by the GenomEast platform of IGBMC (Illkirch, France). RNA-seq libraries were generated from 500 ng of total RNA using the TruSeq Stranded mRNA Library Prep Kit and TruSeq RNA Single Indexes kits A and B (Illumina), according to the manufacturer's instructions. The read length was 50 nt. The mean total reads per sample was 59,103,999.

Mapping of the reads was processed with STAR 2.7.3a on the primary assembly of the latest release of the human genome (56) (GRCh38.p13, release 33, PRI version: https://www.gencodegenes.org/human/) with corresponding comprehensive gene annotations. No soft clipping was accepted. Of the reads, 79% mapped once on the genome, 14% multiple times and 6.4% were below the minimum length threshold to map. Reads were counted using htseq-count version 0.11.2 (57) with reverse strand matching (option “stranded reverse”). Differential expression analysis was done with DESeq2 1.24.0 (58) with Benjamini-Hochberg correction for multiple tests on R 3.6.2.

### RT-qPCR.

Total RNA was extracted from cultured cells using the NucleoSpin RNA II Kit (Macherey-Nagel). The RNA obtained was reverse-transcribed to cDNA using the RevertAid H minus First Strand cDNA Kit (Life Technologies) according to the manufacturer’s protocols. Real-time quantitative PCR (qPCR) was performed using the LightCycler 480 SYBR green I Master (Roche) or the Mesa Green qPCR MasterMix Plus for SYBR Assay (Eurogentec) with the primers listed in Table S4. Primers were selected on PrimerBank database (59). Reactions were run in triplicate, and expression was normalized to GAPDH. The expression analysis was performed using the MxPro QPCR software (Agilent).

### ChIP.

ChIP was performed using the Shearing ChIP and OneDay ChIP kits (Diagenode) according to the manufacturer’s instructions. Briefly, cells were sonicated to obtain DNA fragments of 200 to 500 bp. Sheared chromatin was immunoprecipitated with antibodies against the following proteins/tags: HA (Abcam, ref. ab9110), p73 (Abcam, ref. ab215038), E2F4 (Santa Cruz Biotechnology, ref. SC-398543X), and p130 (Cell Signaling, ref. 13610S). 10% of the sheared chromatin was kept as the input for the ChIP.

Immunoprecipitated chromatin has been analyzed by q-PCR using the LightCycler 480 SYBR green I Master (Roche) on a LightCycler 96 Instrument or the Mesa Green qPCR MasterMix Plus for SYBR Assay (Eurogentec) on a Stratagene Mx3005P Multiplex Quantitative Real-Time PCR System. The sequences of primers used for qPCR are described in Table S4. Primers surrounding the target region were checked for specificity using the NCBI Primer designing tool. ChIP qPCR results were analyzed by evaluating signal of enrichment over noise normalized to Input.

### β-galactosidase and SAHF staining for senescence analyses.

38HK cells were transduced with p19INK4d and cultured for 72 h. The NIH 3T3 feeder layer was removed with PBS/EDTA from 38HK cultures prior to senescence analyses.

Senescence was assessed using the Senescence β-Galactosidase Staining Kit at pH 6 following the manufacturer’s instructions (Cell Signaling Technology). For SAHF staining, 38HK cells were layered on slides coated with polylysine and fixed in 4% paraformaldehyde in PBS (pH 7.4) for 15 min at room temperature, and permeabilized with 0.1% Triton X-100 in PBS for 15 min (60). Cells were incubated with H3K9me3 antibody (abcam; ab1220) for 2 h at room temperature, followed by incubation with Alexa Fluor 555-conjugated secondary antibody for 1 h at room temperature, and mounted using Vectashield Antifade Mounting Medium with DAPI. The slides were visualized using a Nikon Eclipse Ti wide-field inverted fluorescence video microscope. The images captured were analyzed by NIS-Element software from Nikon.

### Quantification and statistical analysis.

Quantification of protein levels from Western blot bands was done using the Evolution-Capt Edge software (Vilber) or ImageLab software (Bio-Rad). The data presented are expressed as means ± SD. *P* values are calculated using unpaired Student’s *t* test.

### Data availability.

The mass spectrometry proteomics data have been deposited to the ProteomeXchange Consortium via the PRIDE (61) partner repository with the data set identifier PXD022947. The RNAseq analyses have been deposited to the GEO database (62) with the identifier GSE162816. Original data files for Western blot analyses are deposited on the public repository Mendeley Data (https://doi.org/10.17632/rpw2j5fzj4.1).
