# Supplementary figures and images for: The E2F4/p130 Repressor Complex Cooperates with Oncogenic ΔNp73α To Inhibit Gene Expression in Human Papillomavirus 38 E6/E7-Transformed Keratinocytes and in Cancer Cells
Source: mSphere. 2023 Mar 8;8(2):e00056-23. doi: 10.1128/msphere.00056-23 (PMC10117100; doi:10.1128/msphere.00056-23)

**A**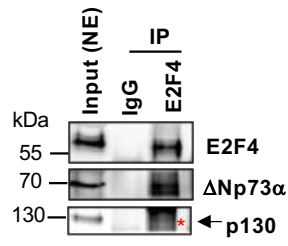**B**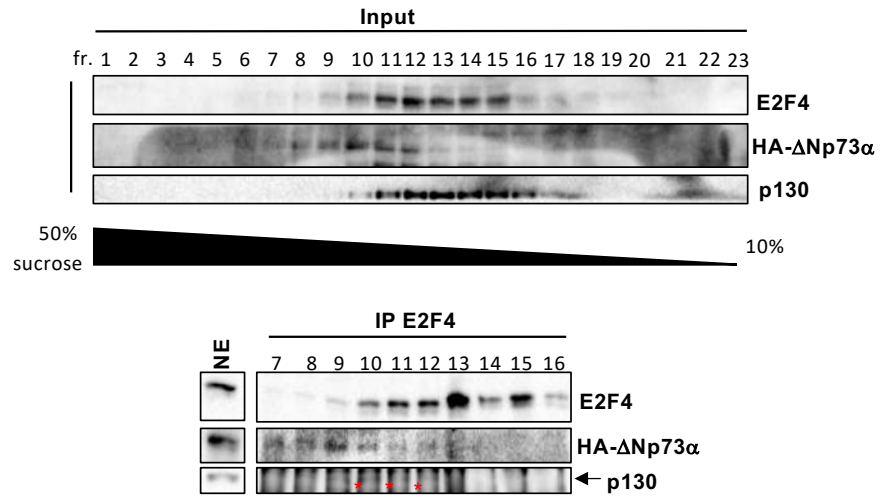**C**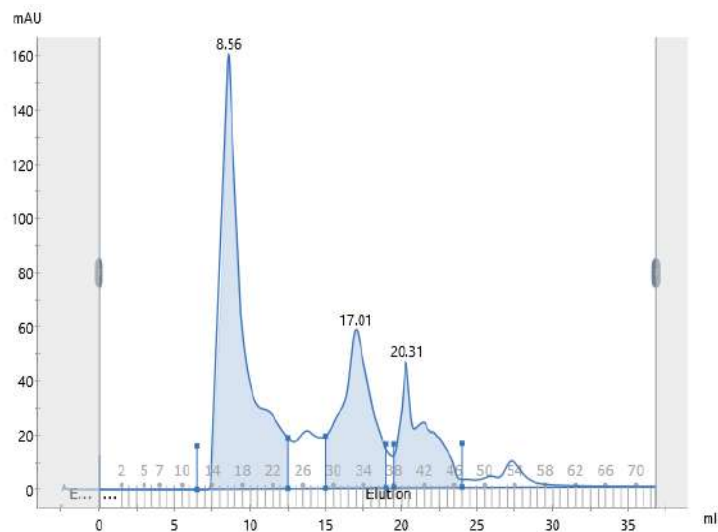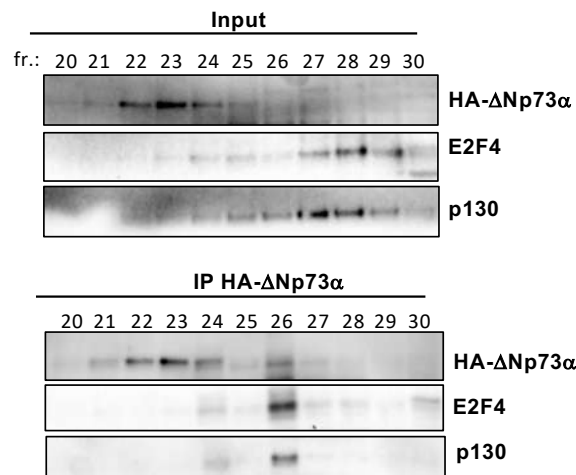

Supplement: FIG S1 [file msphere.00056-23-s0001.pdf]

**A**

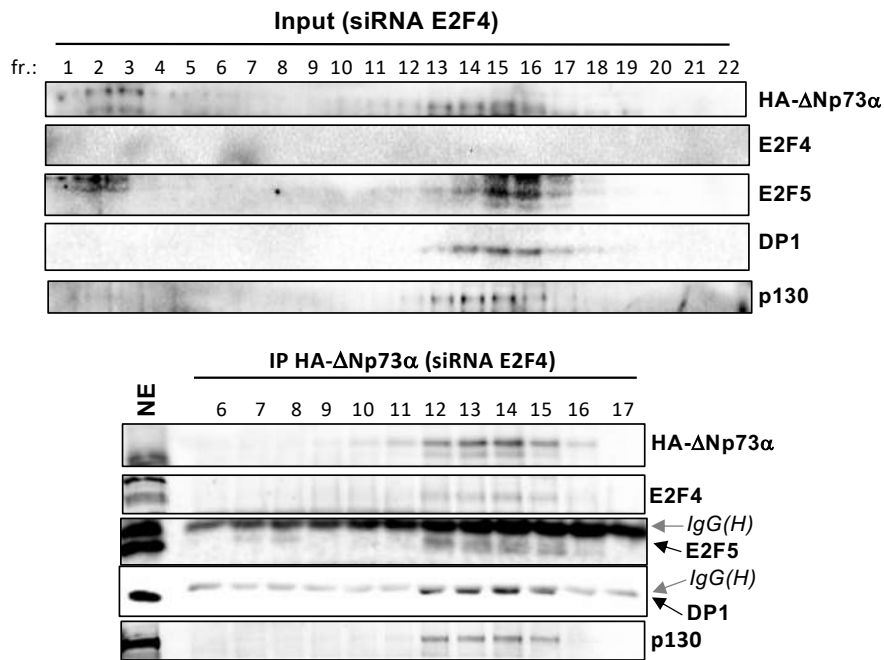

**B**

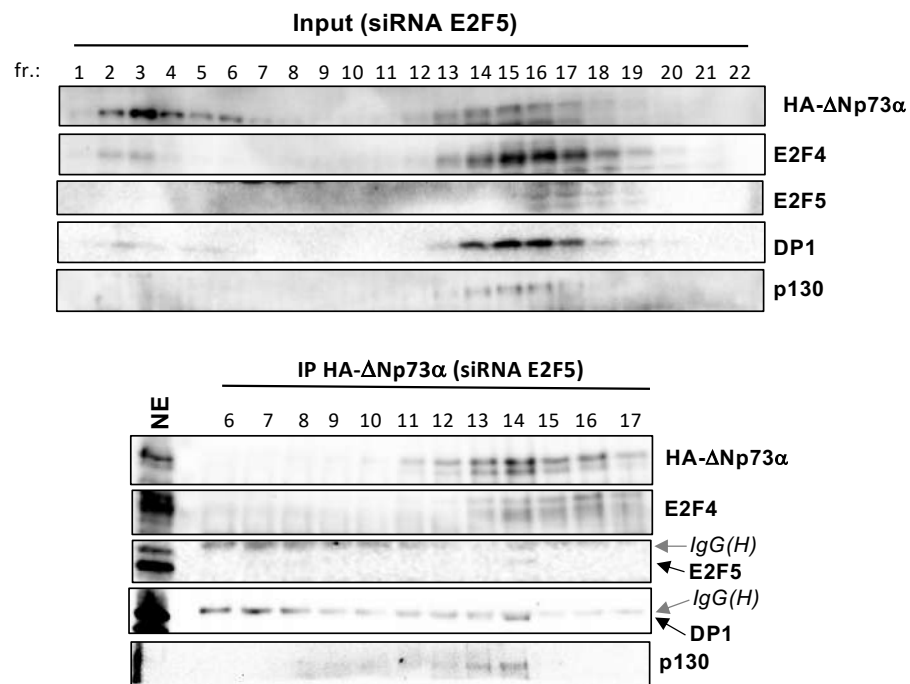

**C**

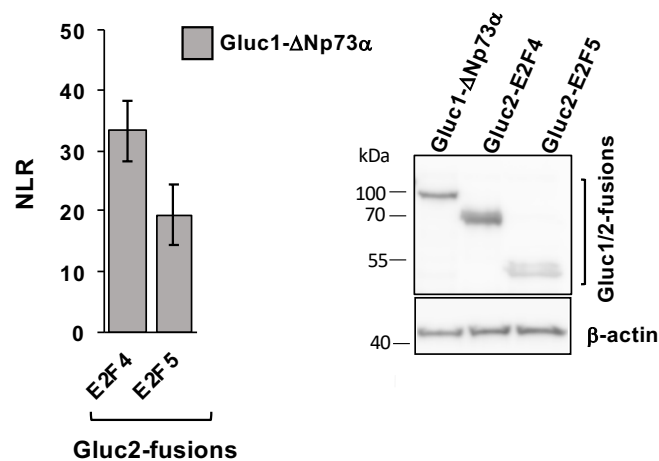

Supplement: FIG S2 [file msphere.00056-23-s0002.pdf]

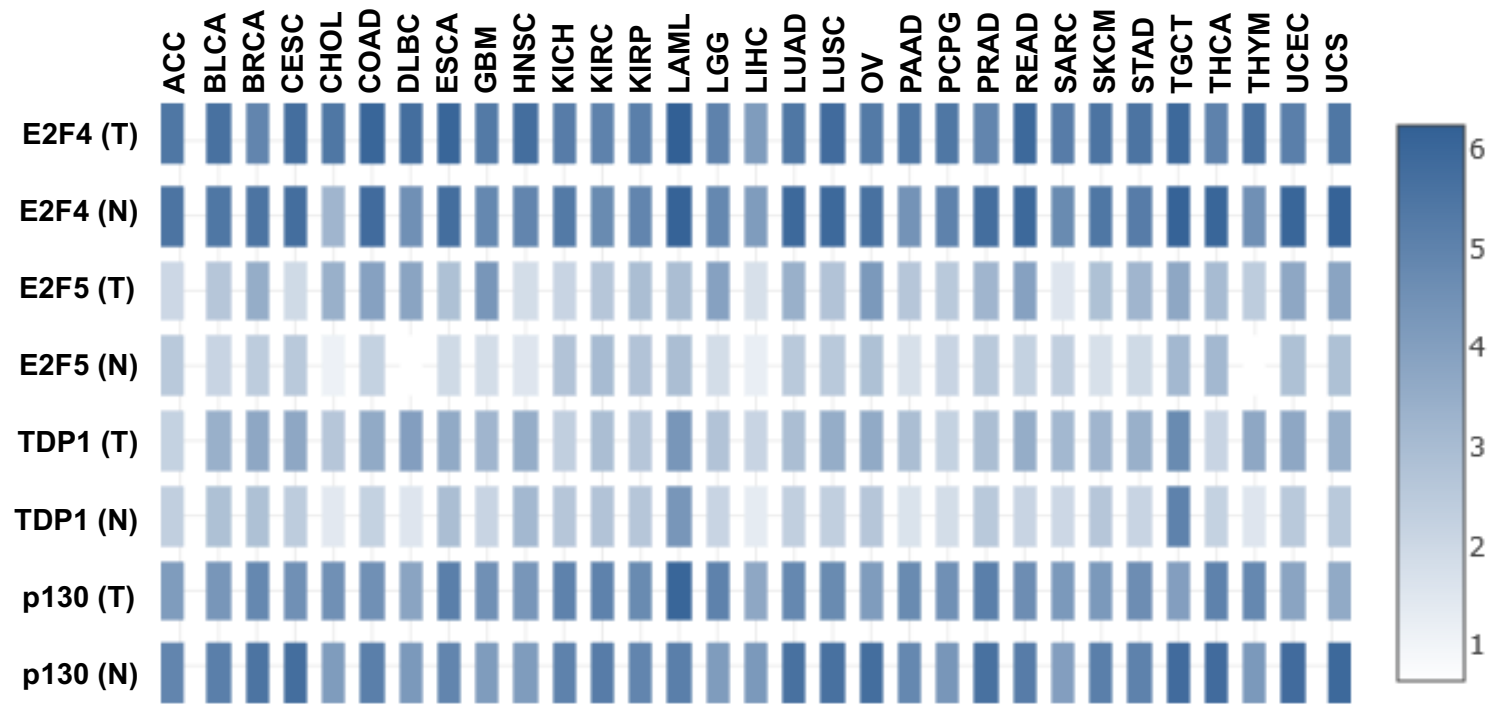

Supplement: FIG S3 [file msphere.00056-23-s0003.pdf]

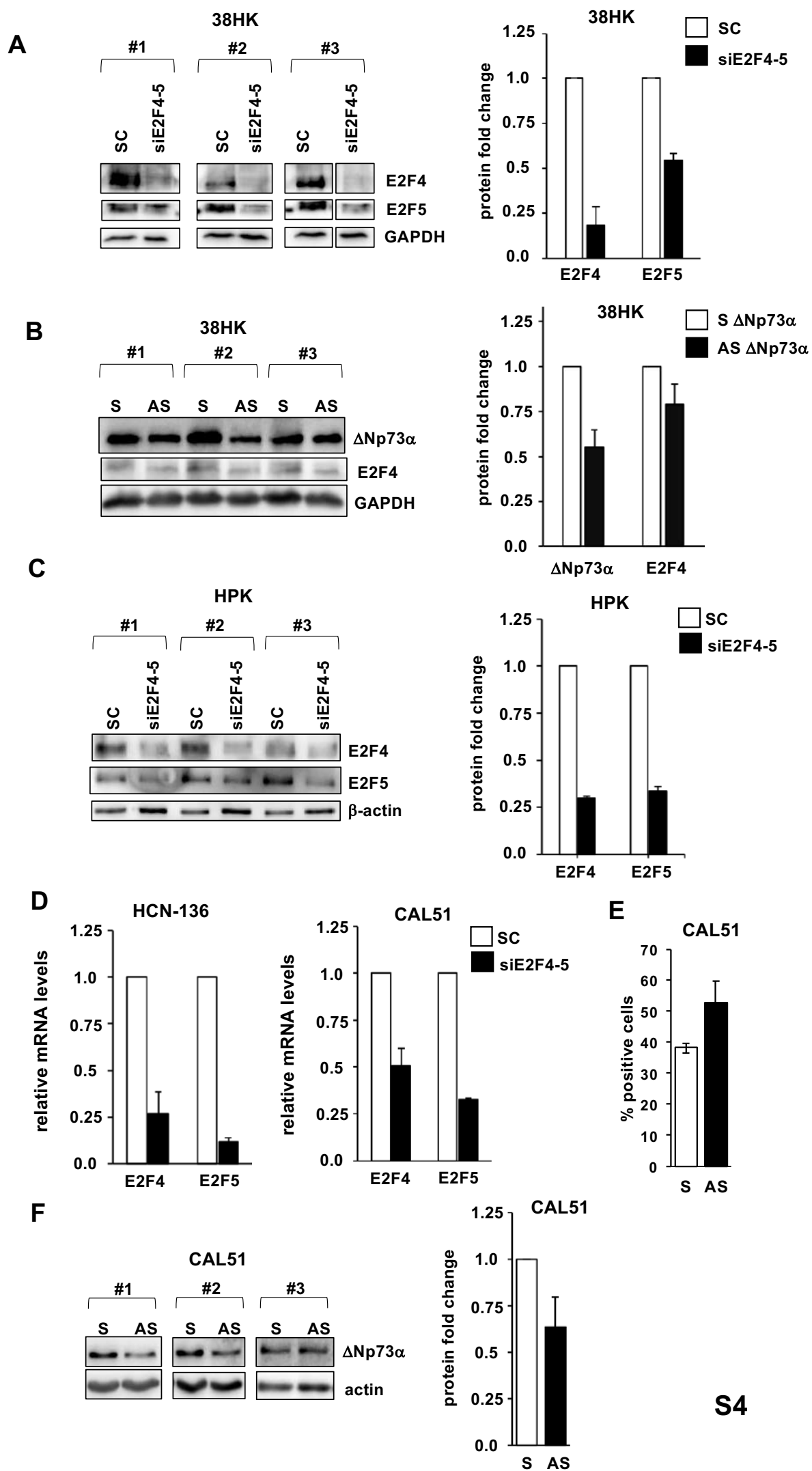

Supplement: FIG S4 [file msphere.00056-23-s0004.pdf]

A

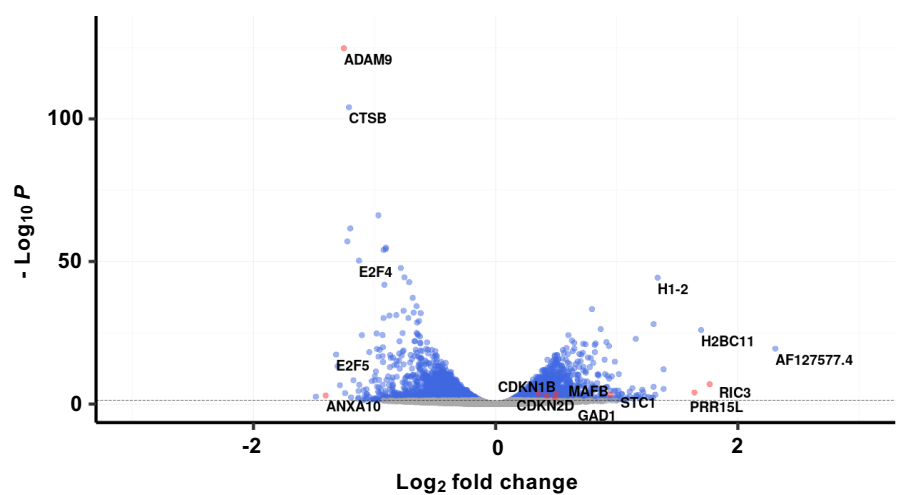

B

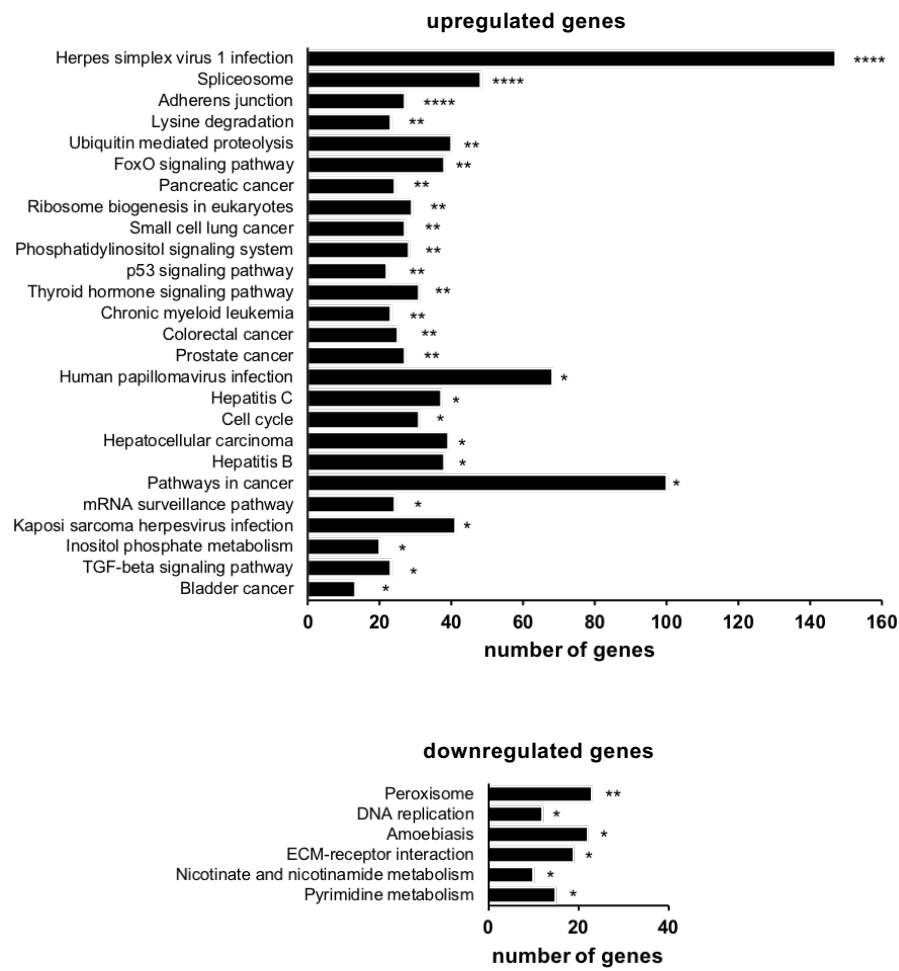

Supplement: FIG S5 [file msphere.00056-23-s0005.pdf]

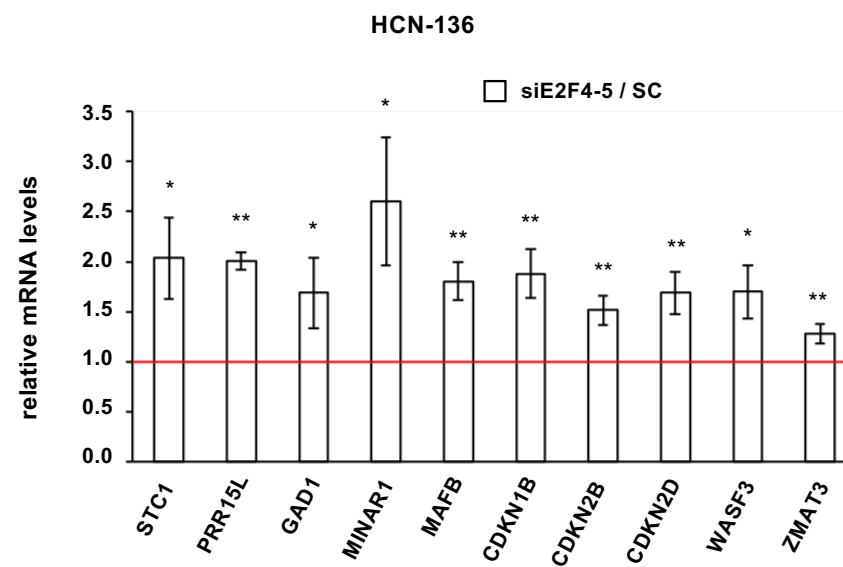

Supplement: FIG S6 [file msphere.00056-23-s0006.pdf]
