# Supplementary material for: The E2F4/p130 Repressor Complex Cooperates with Oncogenic ΔNp73α To Inhibit Gene Expression in Human Papillomavirus 38 E6/E7-Transformed Keratinocytes and in Cancer Cells
Source: mSphere. 2023 Mar 8;8(2):e00056-23. doi: 10.1128/msphere.00056-23 (PMC10117100; doi:10.1128/msphere.00056-23)
Supplement: TABLE S3 [file msphere.00056-23-s0009.docx]

| GENE | Rescue upon E2F4-5 KD  (RT-qPCR) | Rescue upon ΔNp73α KD  (RT-qPCR) | TP53/TP73 RE |
| --- | --- | --- | --- |
| STC1 | + | + | + |
| PRR15L | + | + | + |
| RIC3 | + | + | + |
| GAD1 | + | + | - |
| MAFB | + | + | - |
| MINAR1 | + | + | + |
| ANKRD22 | + | - | + |
| RBM44 | + | - | + |
| TEX15 | + | - | - |
| CCNG1 | + | - | + |
| CDKN1B | + | + | + |
| CDKN2B | + | + | + |
| CDKN2D | + | + | - |
| ZMAT3 | + | +/- | + |
| PTEN | + | - | - |
| WASF3 | + | +/- | + |
| BCL2L11 | - | +/- | + |
| ZNF540 | -/+ | -/+ | - |
| SFMBT2 | -/+ | -/+ | + |
| NR1D2 | - | not tested | + |
| IL20RA | - | not tested | + |
| LANCL3 | - | not tested | + |
| MDM2 | - | not tested | + |

**Supplementary Table S3. Effects of E2F4-5 and ΔNp73α knockdown on the expression of selected genes in 38HK**
